# A specific low-density neutrophil population correlates with hypercoagulation and disease severity in hospitalized COVID-19 patients

**DOI:** 10.1172/jci.insight.148435

**Published:** 2021-05-10

**Authors:** Samantha M. Morrissey, Anne E. Geller, Xiaoling Hu, David Tieri, Chuanlin Ding, Christopher K. Klaes, Elizabeth A. Cooke, Matthew R. Woeste, Zachary C. Martin, Oscar Chen, Sarah E. Bush, Huang-ge Zhang, Rodrigo Cavallazzi, Sean P. Clifford, James Chen, Smita Ghare, Shirish S. Barve, Lu Cai, Maiying Kong, Eric C. Rouchka, Kenneth R. McLeish, Silvia M. Uriarte, Corey T. Watson, Jiapeng Huang, Jun Yan

**Affiliations:** 1 Department of Microbiology and Immunology,; 2 Division of Immunotherapy, the Hiram C. Polk, Jr., MD, Department of Surgery, Immuno-Oncology Program, James Graham Brown Cancer Center,; 3 Department of Biochemistry and Molecular Genetics,; 4 Department of Oral Immunology and Infectious Diseases, School of Dentistry,; 5 Department of Anesthesiology and Perioperative Medicine,; 6 Division of Pulmonary, Critical Care and Sleep Disorders, Department of Medicine,; 7 University of Louisville Hepatobiology and Toxicology Center, Departments of Medicine and Pharmacology & Toxicology,; 8 Pediatric Research Institute, Department of Pediatrics,; 9 Department of Bioinformatics and Biostatistics,; 10 Department of Computer Science and Engineering, and; 11 Division of Nephrology and Hypertension, Department of Medicine, University of Louisville, Louisville, Kentucky, USA.

**Keywords:** COVID-19, Immunology, Coagulation, Neutrophils

## Abstract

SARS coronavirus 2 (SARS-CoV-2) is a novel viral pathogen that causes a clinical disease called coronavirus disease 2019 (COVID-19). Although most COVID-19 cases are asymptomatic or involve mild upper respiratory tract symptoms, a significant number of patients develop severe or critical disease. Patients with severe COVID-19 commonly present with viral pneumonia that may progress to life-threatening acute respiratory distress syndrome (ARDS). Patients with COVID-19 are also predisposed to venous and arterial thromboses that are associated with a poorer prognosis. The present study identified the emergence of a low-density inflammatory neutrophil (LDN) population expressing intermediate levels of CD16 (CD16^Int^) in patients with COVID-19. These cells demonstrated proinflammatory gene signatures, activated platelets, spontaneously formed neutrophil extracellular traps, and enhanced phagocytic capacity and cytokine production. Strikingly, CD16^Int^ neutrophils were also the major immune cells within the bronchoalveolar lavage fluid, exhibiting increased CXCR3 but loss of CD44 and CD38 expression. The percentage of circulating CD16^Int^ LDNs was associated with D-dimer, ferritin, and systemic IL-6 and TNF-α levels and changed over time with altered disease status. Our data suggest that the CD16^Int^ LDN subset contributes to COVID-19–associated coagulopathy, systemic inflammation, and ARDS. The frequency of that LDN subset in the circulation could serve as an adjunct clinical marker to monitor disease status and progression.

## Graphical abstract

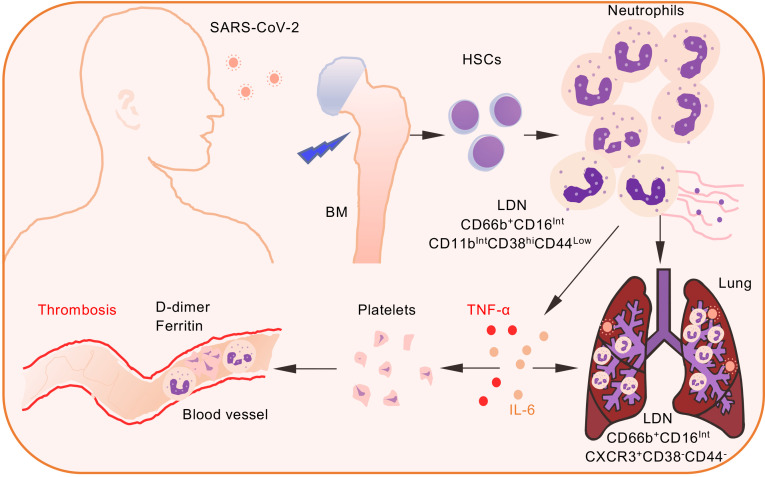

## Introduction

In December 2019, a novel viral pathogen, SARS coronavirus 2 (SARS-CoV-2) emerged that causes a clinical disease called coronavirus disease 2019 (COVID-19). Although a majority of COVID-19 cases are asymptomatic or involve mild upper respiratory tract symptoms, studies early in the pandemic reported up to 20% of patients develop severe or critical disease (1). Patients with severe COVID-19 commonly develop lower respiratory tract disease due to viral pneumonia that progresses to life-threatening acute respiratory distress syndrome (ARDS) in 12% to 25% of hospitalized patients (2, 3). Fluid accumulation in the lungs that is pathognomonic for ARDS results from a combination of virally induced lung injury and the rapid influx of immune cells to fight the infection (4). These recruited inflammatory cells are often in a hyperactivated state associated with a phenomenon known as cytokine storm (5). A variety of cytokines are elevated during cytokine storm, including IL-6, IL-1β, and TNF-α (6). Levels of all 3 cytokines are elevated in the peripheral blood of COVID-19 patients (7–9). Persistently high levels of cytokines are associated with increased risk of vascular hyperpermeability, multiorgan failure, and death (10).

In addition to significant pulmonary complications, patients with severe COVID-19 have a notable coagulopathy (11, 12). Up to 60% of critically ill COVID-19 patients develop COVID-19–associated coagulopathy (CAC), manifested by increased D-dimer levels, unchanged or modestly decreased platelet count, decreased prothrombin time or partial thromboplastin time, and an increased risk of microvascular or macrovascular thrombosis (13–16). Based on the association of CAC with worse patient outcomes, high-intensity thromboprophylaxis or therapeutic anticoagulation were proposed for severely ill or intubated COVID-19 patients (11, 12). Although not yet peer reviewed, preliminary results from the REMAP/ATTACC/ACTIV4a trials suggest a benefit of therapeutic anticoagulation in moderately ill COVID-19 patients but not in critically ill patients (17). In addition, intermediate-dose prophylactic anticoagulation did not lead to a significant difference in the primary outcomes in patients with severe COVID-19 compared with standard dose prophylactic anticoagulation (18). Thus, empiric intensification of anticoagulation in critically ill COVID-19 patients should be pursued with caution (19). Excessive inflammation, platelet activation, neutrophil extracellular trap (NET) formation, and endothelial dysfunction are factors postulated to induce CAC (14, 20, 21). In addition, IL-6 and TNF-α alter platelet activation and/or the coagulation cascade, which may contribute to CAC (22, 23). However, the cellular and molecular pathophysiology of CAC remains to be fully elucidated.

Evidence increasingly supports a role for neutrophils in ARDS and vascular thrombosis occurring in patients with severe COVID-19 (24–27). Severe COVID-19 patients have a distinct immunological phenotype characterized by lymphopenia and neutrophilia, and an increased neutrophil to lymphocyte ratio (NLR) is associated with high D-dimer levels, enhanced vascular thrombosis, and worse clinical outcomes (7). Lung specimens at autopsy showed a marked infiltration of neutrophils into the lung tissue (27–29). Neutrophils are thought to be recruited to the lungs to aid in the clearance of the viral pathogens through phagocytosis, generation of ROS, and cytotoxic granule release (30, 31). However, prolonged neutrophil activation associated with delayed apoptosis and increased NET formation is linked to alveolar damage and adverse outcomes in patients with H1N1 influenza (32, 33). NET formation is postulated to play a prominent role in COVID-19 intravascular coagulation (28).

The current study was initiated to examine the possibility that the neutrophils are significantly contributing to the coagulopathy in hospitalized COVID-19 patients. We found a marked increase in the CD66b^+^ low-density neutrophils (LDNs) within the PBMC compartment of patients with COVID-19. Within the severe COVID-19 patient cohort, we saw the emergence of a significant population of LDNs expressing intermediate levels of CD16 (CD16^Int^ LDNs). A similar population of neutrophils predominated in the bronchoalveolar lavage (BAL) fluid. Transcriptomic profiling and functional analysis of CD16^Int^ LDNs revealed a proinflammatory phenotype, suggesting that CD16^Int^ LDNs significantly contribute to immunothrombosis and systemic inflammation in hospitalized COVID-19 patients.

## Results

### Clinical characteristics of patients with COVID-19.

In our study, a total of 53 patients who tested positive for SARS-CoV-2 by nasopharyngeal swab were screened and recruited. Additionally, 9 patients with similar comorbidities but negative for SARS-CoV-2 and 6 healthy donors were recruited as controls. The study participant demographics and summary of clinical information are shown in Supplemental Table 1; supplemental material available online with this article; https://doi.org/10.1172/jci.insight.148435DS1 For the neutrophil immunophenotyping study, 10 patients were initially enrolled in the severe category, as defined by necessity of mechanical ventilation within the intensive care unit (ICU), and 21 were initially enrolled in the moderate group, as patients were admitted to the hospital but were not on mechanical ventilation. Of note, 2 of the originally enrolled moderate patients progressed to the severe category; 3 severe patients improved to be classified as moderate during the course of our study. Those patients were counted as individual patients within their original or secondary groups depending on the classification on the day blood was obtained. The study participant demographics and summary of clinical information on immunophenotyped patients are shown in Supplemental Table 2. Peripheral blood samples were obtained daily from either a venous or arterial line for patients with severe Illness, whereas samples from moderate patients were obtained from a venous line approximately every 2 to 3 days.

### LDNs are significantly increased in hospitalized COVID-19 patients, and CD16^Int^ LDNs are specifically expanded by SARS-CoV-2 infection.

Previous studies indicated a dominant neutrophilia in severe COVID-19 patients (7). We confirmed this finding from patient whole blood complete blood count (CBC) reports. We first partitioned all serial blood draws from each patient based on whether they were classified as moderate or severe on the day blood was obtained, and then averaged data by classification for each patient. These data demonstrated that there was an approximately 10% increase in neutrophil percentage in the peripheral blood of patients at severe time points compared with moderate time points and a 30% increase in neutrophil percentage over what was observed in healthy donors (Supplemental Figure 1A). Conversely, the overall lymphocyte percentage in patients at severe time points was decreased compared with the moderate time points and healthy donors. Interestingly, comorbidity control patients also showed a decreased lymphocyte percentage (Supplemental Figure 1A). viSNE analysis of the overall CD3^+^ T cells and CD4^+^ and CD8^+^ T cell subsets showed a decreasing population size in patients with moderate and severe COVID-19 as well as in comorbidity control patients compared with healthy donors (Supplemental Figure 1B). Our data showing an increased NLR within our severe cohort agrees with previously published reports (34, 35).

**Figure 1:**
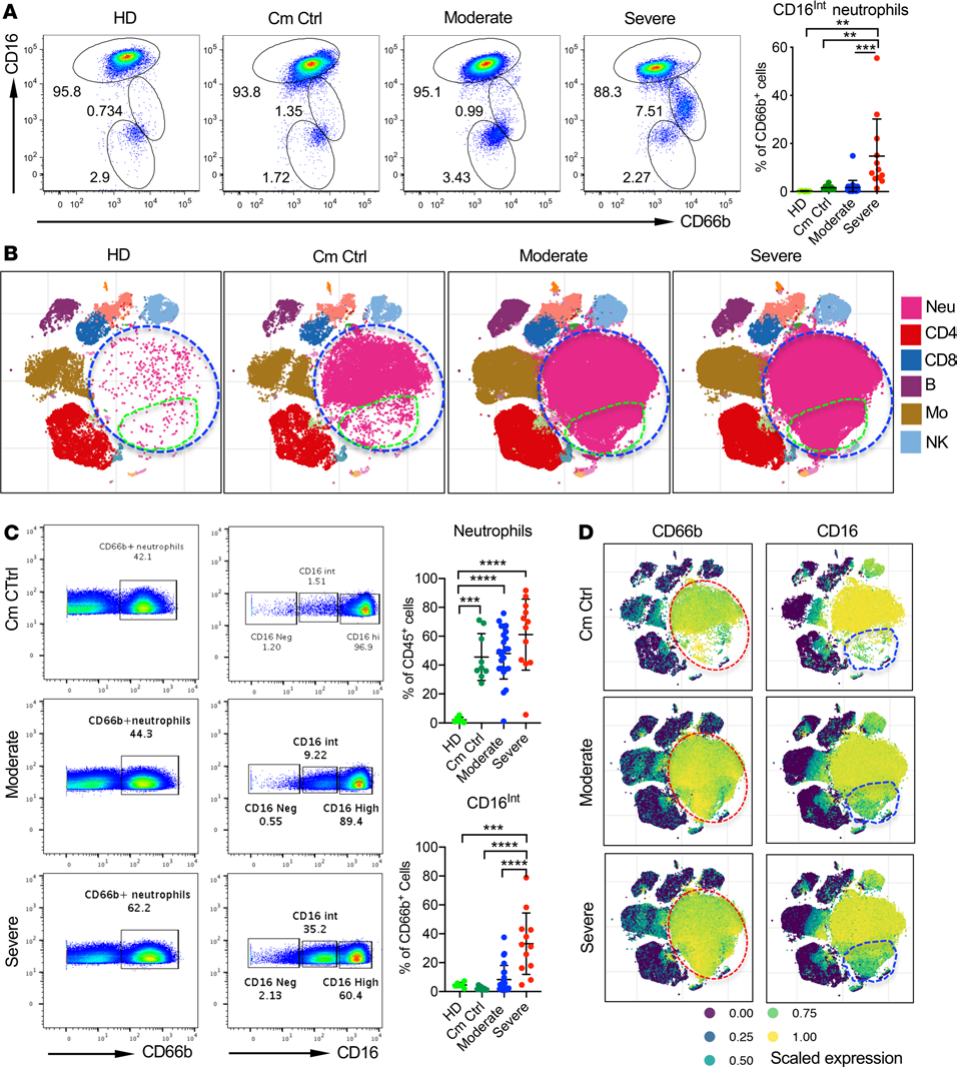
The identification of a CD16 intermediate low-density neutrophil population in COVID-19 patients. (**A**) The averaged percentage of CD16-negative (CD16^Neg^), CD16 intermediate (CD16^Int^), and CD16 high (CD16^hi^) neutrophils from serially drawn whole blood samples among healthy donors (HD, *n* = 6), comorbid control patients (Cm Ctrl, *n* = 9), and moderate (*n* = 24) and severe (*n* = 12) COVID-19 patients. Cells were gated on the CD45^+^CD66b^+^ population. Summarized data and representative dot plots are shown. (**B**) Cluster maps for moderate and severe COVID-19 patients compared with HD and Cm Ctrl. The data were generated from CyTOF-based analysis of CD45^+^ PBMCs isolated from peripheral blood. (**C**) Representative dot plots (left) and summarized data (right) showing the overall percentage of CD66b^+^ neutrophils (gated on viable, CD45^+^) and the CD16^Int^ subset as found in Ficoll-isolated PBMCs analyzed using CyTOF mass cytometry in HD, Cm Ctrl, and moderate and severe COVID-19 patients. (**D**) Representative viSNE cluster plots show the CD66b (left) and CD16 (right) expression within the CD45^+^ PBMC populations in Cm Ctrl and patients with moderate and severe COVID-19. Red circles indicate the location of the neutrophil population; blue circles indicate the CD16^Int^ neutrophil population. Data are presented as mean ± SD. *P* values were determined using 1-way ANOVA with multiple comparisons. **P < 0.01, ***P < 0.001, ****P < 0.0001.

Analysis of the neutrophil pool revealed 3 distinct subpopulations within whole blood samples that clustered by CD16^lo^, CD16^Int^, and CD16^hi^ expression. Severe COVID-19 patients showed a marked increase in the CD16^Int^ subset, which was significantly lower in the moderate cohort and comorbidity controls and virtually absent in the healthy donors (Figure 1A). CD16^Int^ neutrophils classically are reported to be LDNs or immature neutrophils (36, 37). Clinically, immature neutrophils are called band cells and are associated with a left shift on a CBC (38). These neutrophils are often mononucleated and smaller than typical neutrophils. Therefore, the presence of these cells in PBMCs isolated by Ficoll gradient separation was examined. Cell lineage cluster analysis from total PBMC populations assessed by CyTOF mass cytometry indeed demonstrated that CD66b^+^ neutrophils (blue circle, Figure 1B) were the most prominent population in COVID-19 patients. Minimal LDNs were seen in PBMC preparations of healthy donors (Figure 1B). We also identified a specific population within the neutrophil cluster that showed significant expansion only in COVID-19 patients (green circle, Figure 1B).

We further examined CD16 expression on neutrophils from the PBMC preparation. Similar to the whole blood samples, LDNs in PBMCs also showed 3 populations based on CD16 expression (Figure 1C). Despite increased overall neutrophils in comorbidity controls, CD16^Int^ neutrophils were only increased in moderate and severe COVID-19 patients (Figure 1C), suggesting that SARS-CoV-2 infection specifically drives expansion of this subset of neutrophils. Cluster analysis of isolated PBMCs from a single blood draw from each participant indicated a predominant neutrophil population within the CD45^+^ compartment in the severe and moderate COVID-19 cohorts compared with comorbidity control patients (red circle, Figure 1D, left panel). Additionally, there was a subset of the neutrophil population expressing intermediate CD16 levels in COVID-19 patients, which was almost absent in comorbidity control patients (blue circle, Figure 1D, right panel).

### Phenotypic characterization of CD16^Int^ LDNs.

**Figure 2:**
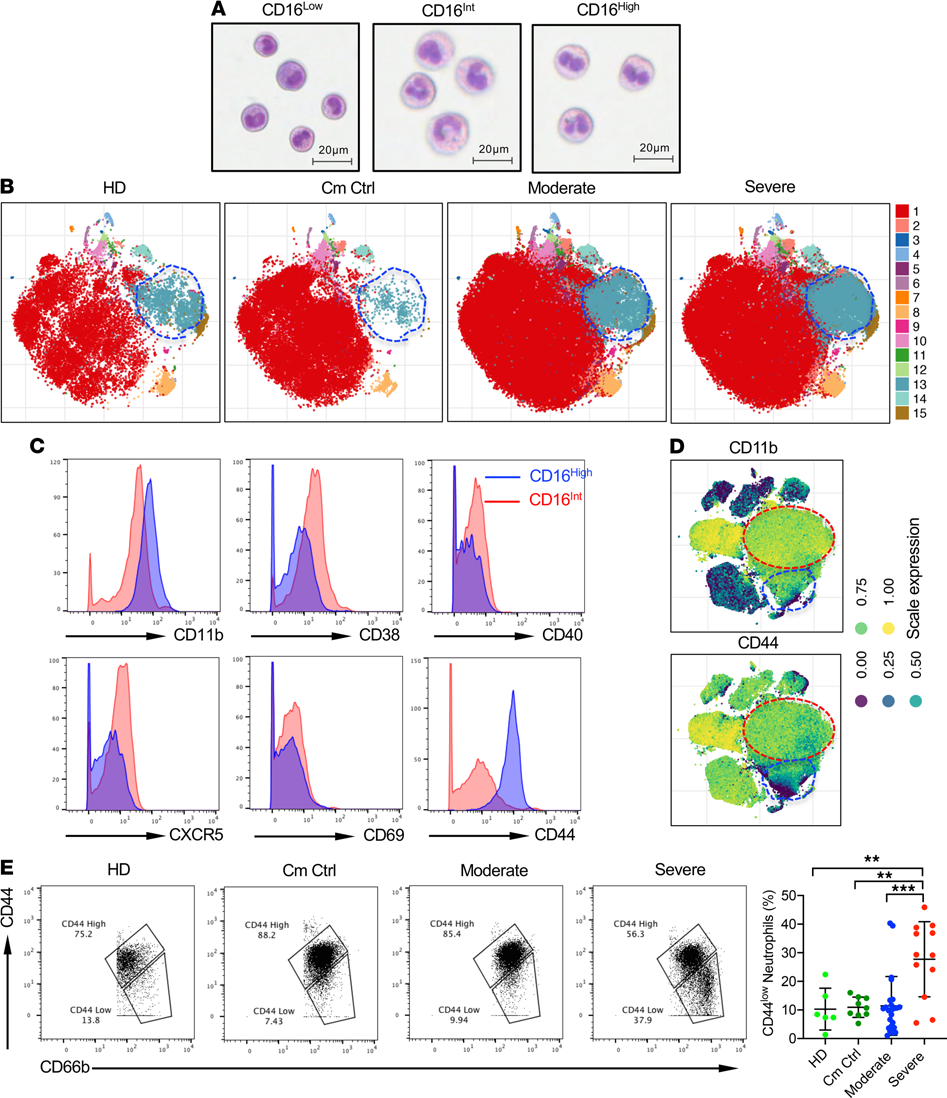
Phenotypic characteristics of neutrophil populations. (**A**) Wright-Giemsa staining of sorted CD66b^+^ CD16^Neg^ (left), CD16^Int^ (middle), and CD16^hi^ (right) populations show different stages of neutrophil maturation. (**B**) Representative cluster maps of CD66b^+^ neutrophil subsets in moderate and severe COVID-19 patients compared with Cm Ctrl and HD groups. (**C**) Representative histograms showing indicated surface molecule expression levels on CD16^hi^ (blue) and CD16^Int^ (red) LDNs. Cells were gated on the viable CD66b^+^ population. (**D**) viSNE cluster plots highlight the expression of CD11b and CD44 in the CD16^Int^ (blue circle) and CD16^hi^ (red circle) neutrophil populations. (**E**) Using mass cytometry, CD44 expression on CD66b^+^ neutrophils from HD (*n* = 6), Cm Ctrl (*n* = 9), and moderate (*n* = 24) and severe (*n* = 12) COVID-19 patients is shown. Representative dot plots and summarized data are shown. Data are presented as mean ± SD. *P* values were determined using 1-way ANOVA with multiple comparisons. **P < 0.01, ***P < 0.001.

Maturation of neutrophils from hematopoietic stem cells is identified by stages with distinct morphological characteristics (39). We performed Wright-Giemsa staining to determine whether the 3 CD16 populations of neutrophils were actually neutrophils in the later 3 stages of development: myelocyte, metamyelocyte (band cell), and granulocyte (mature neutrophil). Figure 2A clearly shows that the CD16^lo^ cells were basophilic myelocytes with an ovoid nucleus, the CD16^Int^ cells contained a large number of band cells with the characteristic band-shaped nucleus, and the CD16^hi^ cells were segmented, mature neutrophils. It is worth noting, however, that the mature CD16^hi^ neutrophils were typically bilobed rather than hypersegmented and closely resembled pseudo-Pelger–Huet cells described in other severe infections like influenza A, tuberculosis, and HIV (40).

Next, we explored differential surface marker expression on the different CD16^+^ neutrophil subsets from COVID-19 patients. A cluster analysis of the overall CD66b^+^ neutrophil population showed an increased prevalence of cluster 13 in the COVID-19 patient cohorts compared with comorbidity controls and healthy donors (Figure 2B, blue circle). The single cell marker expression profiles (Supplemental Figure 2) revealed that cluster 13 showed decreased expression of CD44, CD16, and CD11b. Tracking the neutrophil clusters in serial blood draws over 5 days from different types of patients revealed the dynamic nature of neutrophil pools in COVID-19 infection (Supplemental Figure 3, A and B). In the severe patient, the light blue population (cluster 4, black circle) increased over time while all the other clusters remained similar. For the moderate patient, the majority of clusters remained stable over time. The patient who was initially enrolled in the severe cohort, but improved to moderate by day 5, had a profound decrease in cluster 5 (red circle) over time. Conversely, in the patient that transitioned from moderate to severe, the light blue (cluster 4) and purple (cluster 5) clusters increased over time, which was consistent with the change in disease severity.

Given that the profile of neutrophil clusters associates with disease status, we next determined specific surface marker phenotypes for the different CD16 neutrophil clusters using mass cytometry. As compared with CD16^hi^ LDNs, CD16^Int^ LDNs expressed an intermediate level of CD11b and an elevated level of CD38, CD40, CXCR5, and CD69, suggesting a more activated phenotype (Figure 2C). In addition, CD16^Int^ LDNs showed markedly downregulated CD44 expression (Figure 2C). Cluster analysis revealed that the CD16^Int^ LDN cluster (blue circle) indeed showed decreased CD11b and CD44 expression compared with the CD16^hi^ LDN cluster (red circle, Figure 2D). CD44 is an adhesion receptor for extracellular matrix that has been associated with neutrophilic lung inflammation in bacterial pneumonia. Consistent with the observation that decreased surface expression of CD44 resulted in increased accumulation of neutrophils in the lungs of *E*. *coli*–infected mice (41), neutrophils from severe patients had the lowest expression of CD44 (Figure 2E).

### CD16^Int^ LDNs exhibit proinflammatory gene signatures with increased phagocytic capacity and spontaneous NET formation.

**Figure 3:**
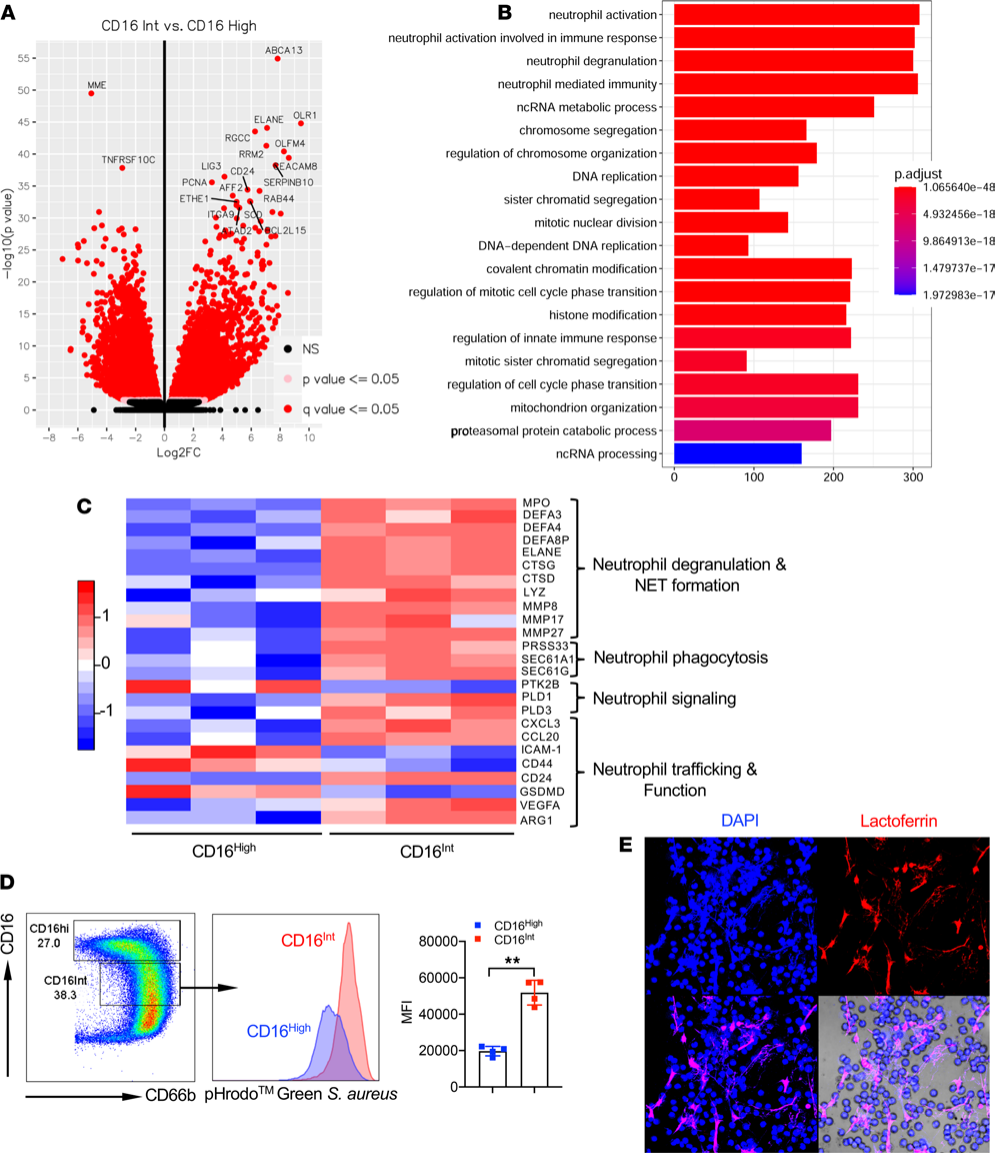
CD16^Int^ LDNs exhibit proinflammatory gene signatures with functionally active phenotype. (**A**) Volcano plot shows differentially expressed genes (DEGs) between CD16^Int^ and CD16^hi^ LDN. (**B**) Top 20 enriched gene ontology biological pathway categories for CD16^Int^ versus CD16^hi^ LDNs from severe COVID-19 patients. (**C**) The heatmap shows DEGs related to neutrophil degranulation and NET formation, neutrophil phagocytosis, neutrophil signaling, and neutrophil trafficking and function between CD16^hi^ and CD16^Int^ LDNs. (**D**) The phagocytic capacity of CD16^hi^ and CD16^Int^ neutrophils from whole blood in severe COVID-19 patients (*n* = 4) was assessed using a pHrodo Green *S*. *aureus* BioParticles phagocytosis assay. Gating strategy, representative histogram, and summarized MFI data are shown. **P < 0.01 (Student’s *t* test). (**E**) Representative confocal image of spontaneous NET formation from sorted CD16^Int^ LDNs. Anti-human lactoferrin (shown in red) and neutrophil DNA stained with DAPI (shown in blue) merge image shows NET characteristic structures. Original magnification, ×60. Data are presented as mean ± SD.

To define gene signatures of LDN subsets in COVID-19 patients, we sorted CD16^hi^ and CD16^Int^ LDNs from 3 severe COVID-19 patients. Normal-density neutrophils (NDNs) were obtained from healthy donors. RNA was extracted from each neutrophil population and RNA-Seq was performed. Principal component analysis (PCA) showed striking differential aggregations among the 3 populations (Supplemental Figure 4A). We focused our comparison on CD16^hi^ and CD16^Int^ LDNs from COVID-19 patients. A total of 6387 differentially expressed genes (DEGs) were observed comparing CD16^Int^ to CD16^hi^ LDNs (3116 upregulated DEGs and 3271 downregulated DEGs, Figure 3A). Gene ontology biological pathway analysis showed that the neutrophil activation, neutrophil activation involved in immune response, neutrophil degranulation, and neutrophil-mediated immunity were ranked as the top 4 enriched pathways in these DEGs (Figure 3B). CD16^hi^ and CD16^Int^ LDNs expressed DEGs related to neutrophil activation and neutrophil activation–involved immune responses (Supplemental Figure 4B). Gene set enrichment analysis (GSEA) indicated that genes related to chronic inflammatory response, positive regulation of inflammatory response, positive regulation of myeloid leukocyte–mediated immunity, superoxide generation, positive regulation of leukocyte degranulation, respiratory burst, regulation of neutrophil chemotaxis, and phagocytosis recognition were significantly enriched in CD16^Int^ LDNs compared with CD16^hi^ neutrophils (Supplemental Figure 4C). We specifically compared DEGs related to neutrophil degranulation, NET formation, phagocytosis, signaling, and neutrophil trafficking and function (Figure 3C). Genes related to neutrophil degranulation, NET formation, and neutrophil phagocytosis were uniformly upregulated in CD16^Int^ LDNs. On the other hand, DEGs related to neutrophil trafficking did not show a consistent pattern. CD44 was downregulated, consistent with our flow cytometry data. *VEGFA* and arginase 1 were upregulated, whereas gasdermin D was downregulated in the CD16^Int^ LDNs.

Given that the transcriptomic analysis revealed increased expression of phagocytic genes, we next investigated the phagocytic functionality of the neutrophils from COVID-19 patients. Figure 3D shows that CD16^Int^ LDNs had a significantly greater uptake of pHrodo Green *S*. *Aureus* bioparticles than CD16^hi^ neutrophils, suggesting an activated phenotype. Another neutrophil antimicrobial mechanism is the formation of NETs, the extravasation of DNA and protein to form a weblike structure that can trap and kill extracellular pathogens. NET formation also contributes to increased platelet aggregation and coagulation (42). Consistent with upregulation of NET-forming genes in this subset (Figure 3C), we observed that CD16^Int^ LDNs spontaneously formed NETs (Figure 3E). Taken together, our gene expression, protein expression, and functional data indicate that CD16^Int^ LDNs exhibit a proinflammatory phenotype, including enhanced phagocytosis, NET formation, and granule mobilization and altered expression of surface molecules that may regulate their migration into the lung.

### CD16^Int^ LDNs interact with platelets for activation, leading to hypercoagulable state in patients with severe COVID-19.

**Figure 4:**
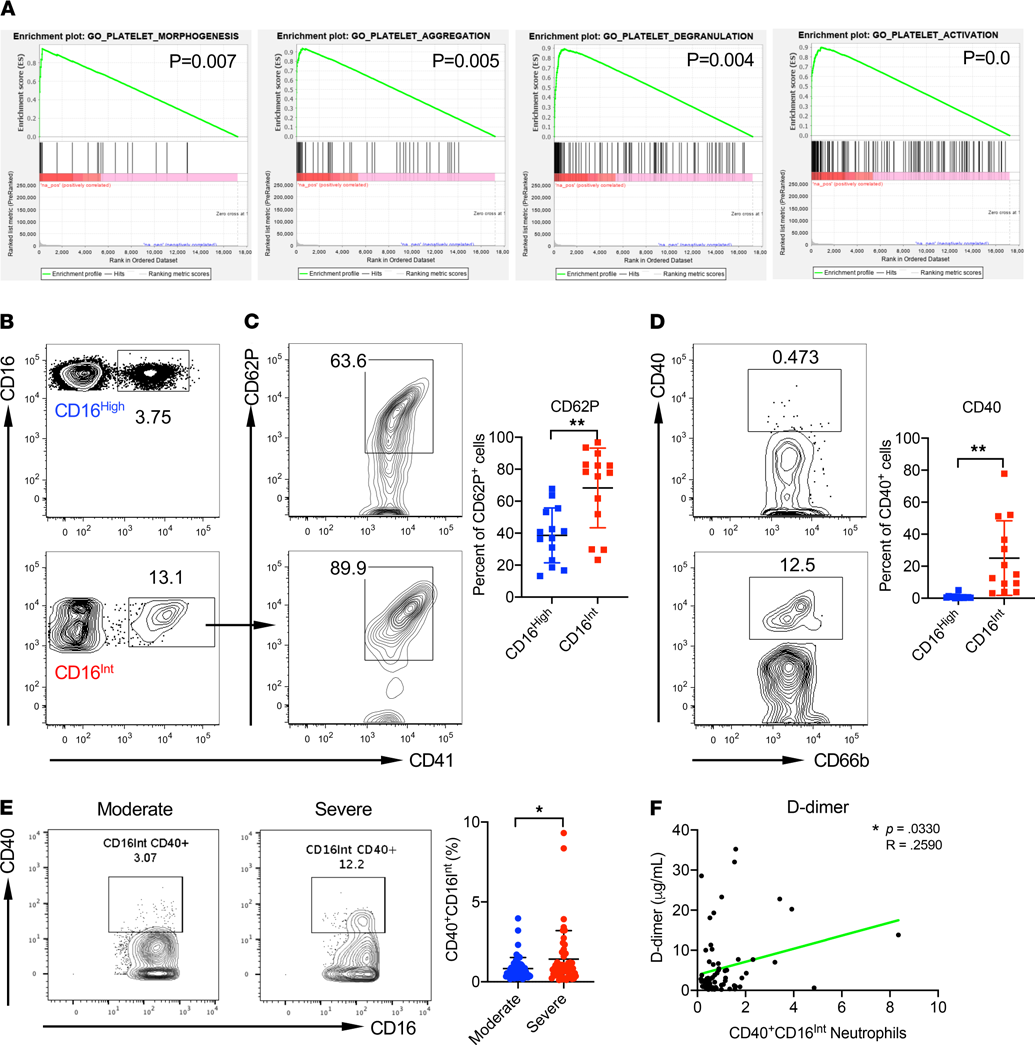
CD16^Int^ LDNs interact with platelets for activation, leading to a hypercoagulable state. (**A**) GSEA shows significantly enriched pathways, including platelet morphogenesis, platelet aggregation, platelet degranulation, and platelet activation in CD16^Int^ LDNs compared with CD16^hi^ LDNs from severe COVID-19 patients (*n* = 3). (**B**) Representative dot plots showing neutrophil-platelet aggregates. Cells were gated on the CD66b^+^ population. (**C**) Gated on neutrophil-platelet aggregates, platelet activation marker CD62P expression levels were measured. Representative dot plots and summarized data are shown. (**D**) Gated on neutrophil-platelet aggregates, CD40 expression levels were detected. Representative dot plots and summarized data are shown. (**E**) CD40 expression levels on CD16^Int^ LDNs from moderate and severe COVID-19 patients. Representative dot plots and summarized data pooled from serial patient draws throughout the course of their hospital admission and grouped according to patient status are shown. A linear mixed-effect model was used to determine significance. *P < 0.05. (**F**) D-dimer levels were correlated with the percentage of CD40^+^CD16^Int^ neutrophils from longitudinal, serial blood draws measured on the day of sample acquisition. Pearson correlations were used to indicate statistical significance. Data are presented as mean ± SD. *P* values were determined using a Student’s *t* test. *P < 0.05, **P < 0.01.

A thrombogenic coagulopathy is associated with COVID-19, and the majority of patients with severe COVID-19 present with elevated D-dimer levels (11). A recent study documented the interaction of NET-forming neutrophils with platelets in pulmonary microthrombi in autopsy specimens and found higher levels of circulating neutrophil-platelet aggregates in patients with COVID-19 (27). Our GSEA showed that genes related to platelet morphogenesis, platelet aggregation, platelet degranulation, and platelet activation were enriched in CD16^Int^ LDNs (Figure 4A). To determine whether LDNs directly interact with platelets, we quantified circulating neutrophil-platelet aggregates in the whole blood samples from additionally recruited COVID-19 patients (Supplemental Table 3). Neutrophil-platelet aggregates were present in both CD16^hi^ and CD16^Int^ neutrophil populations (Figure 4B). To determine the activation status of platelets in those aggregates, expression of CD62P and CD40 by platelets within aggregates was determined by flow cytometry. Both CD62P (Figure 4C) and CD40 (Figure 4D) expression were significantly higher in CD16^Int^ neutrophil-platelet aggregates compared with CD16^hi^ neutrophil-platelet aggregates, indicating that aggregation with CD16^Int^ neutrophils was associated with significantly greater platelet activation.

The CD40/CD40L pathway drives platelet activation and thrombosis. Inhibition of the neutrophil-platelet CD40/CD40L axis with anti-CD40 Ab is reported to significantly reduce pulmonary edema and platelet activation and reduce neutrophil recruitment to the lungs in a mouse model of transfusion-related acute lung injury (43). We found severe COVID-19 patients had significantly more CD40^+^CD16^Int^ LDNs than moderate patients as assessed by flow cytometry (Figure 4E). Moreover, increased CD40 expression by CD16^Int^ LDNs significantly correlated with increased D-dimer levels in severe COVID-19 patients (Figure 4F). These results suggest that CD16^Int^ LDNs may participate in COVID-19 coagulopathy through direct activation and aggregation of platelets.

### CD16^Int^ neutrophils predominate in BAL fluid.

**Figure 5:**
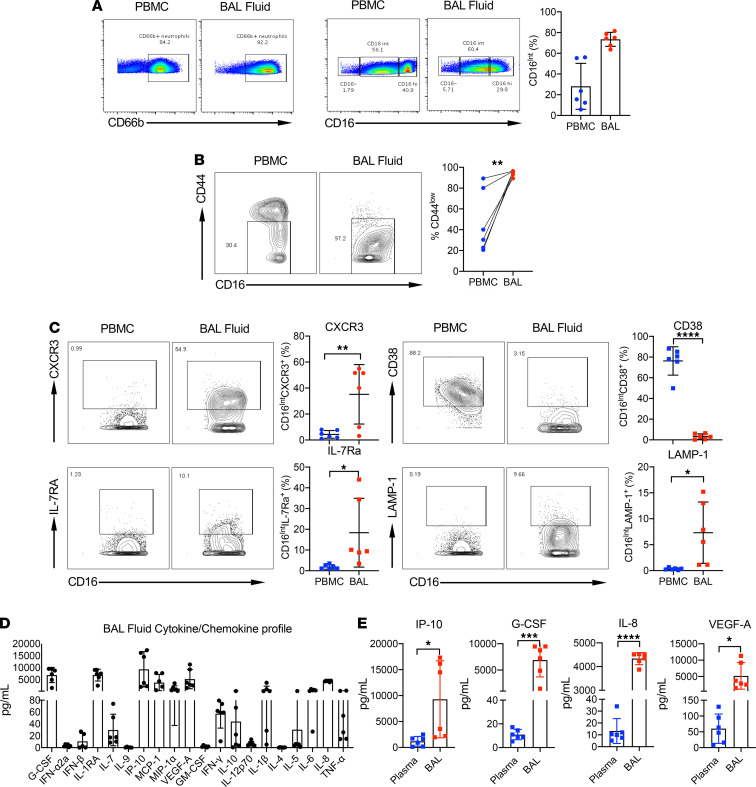
Comparison of PBMC and BAL fluid immune cell populations using CyTOF. (**A**) CD66b^+^ neutrophil populations (left) and CD16-negative, -intermediate, and -high populations (right) were compared in PBMCs and BAL fluid isolated on the same day. (**B**) The expression of CD44 on CD16^Int^ neutrophils was measured in PBMCs and BAL fluid taken on the same day in 3 severe patients. Representative plots (left) and summarized data (right) are shown. (**C**) The expression of CXCR3 and CD38 (top) and IL-7RA and LAMP-1 (bottom) was measured on CD16^Int^ neutrophils in PBMCs and BAL fluid. Representative plots and summarized data are shown. (**D**) Concentration of 20 cytokines/chemokines in the BAL fluid of severe COVID-19 patients (*n* = 6) as measured by U-PLEX assay. (**E**) Concentration of IP-10, G-CSF, IL-8, and VEGF-A in plasma samples versus BAL fluid of severe COVID-19 patients (*n* = 6) as measured by U-PLEX assay. Data are shown as mean ± SD. *P* values were determined using a Student’s *t* test. *P < 0.05, **P < 0.01, ***P < 0.001, ****P < 0.0001.

Neutrophils were observed in alveoli and interstitium of lungs of autopsied COVID-19 patients and were prevalent in BAL fluid from severe COVID-19 patients (24, 27, 28, 44). To determine whether the emergent LDN population we identified in the peripheral blood is associated with increased LDNs in the lungs, we collected BAL fluid from patients with severe COVID-19 (Supplemental Table 4). Neutrophils constituted the major immune cell population within the BAL fluid. Strikingly, CD16^Int^ neutrophils accounted for more than 60% of the total neutrophil population in BAL fluid (Figure 5A). In addition, almost all CD16^Int^ neutrophils in the BAL fluid expressed significantly lower levels of CD44 than peripheral blood CD16^Int^ neutrophils from the same patient (Figure 5B). Comparison of CD16^Int^ neutrophils from peripheral blood and BAL fluid from the same patient identified 4 additional markers that were differentially expressed. CD16^Int^ neutrophils in BAL fluid expressed significantly greater levels of the chemokine receptor CXCR3, whereas CD38 was markedly downregulated (Figure 5C). We also found that CXCR3 expression was higher in CD16^Int^ neutrophils compared with that in the CD16^hi^ population (Supplemental Figure 5A). In contrast, CD44 expression levels were lower in CD16^Int^ neutrophils compared with the CD16^hi^ subset in BAL samples (Supplemental Figure 5A). The expression levels of IL-7Ra and degranulation marker LAMP-1 were also marginally increased in CD16^Int^ neutrophils from BAL fluid (Figure 5C). A previous study showed that CXCR3 is expressed on lung-recruited neutrophils during influenza pneumonia (45). CD38 is an ADP-ribosyl cyclase that controls neutrophil chemotaxis to bacterial chemoattractants (46). Loss of CD38 on CD16^Int^ could contribute to a mechanism where these neutrophils may accumulate in the lungs because of chemokine signaling and without CD38 expression then lack the ability to exit the lungs, leading to neutrophil accumulation.

To evaluate possible stimuli for CD16^Int^ neutrophil trafficking from the periphery to the lung, we assayed chemokines/cytokines in the BAL fluid. High levels of a number of chemokines and cytokines capable of recruiting or activating neutrophils were present in the BAL fluid, including G-CSF, IL-1RA, IP-10, MCP-1, and IL-8 (Figure 5D). Proinflammatory cytokines IL-6 and TNF-α were also present at high concentrations. Consistent with previous studies showing deficient expression of IFN-stimulated genes suggesting defective antiviral immune responses in severe COVID-19 (47, 48), type I IFNs including IFN-α2a and IFN-β were not detectable. Levels of IP-10, G-CSF, IL-8, and VEGFA were significantly increased in the BAL fluid compared with the corresponding plasma samples (Figure 5E). IP-10 (CXCL10) is a chemokine ligand for CXCR3, which as noted above was highly expressed on CD16^Int^ neutrophils from BAL fluid. Collectively, these preliminary data suggest that the CXCL10/CXCR3 axis may participate in CD16^Int^ neutrophil recruitment into the lungs of patients with COVID-19.

### Frequency of CD16^Int^ LDNs is correlated with plasma levels of IL-10, IL-1R, MCP-1, and MIP-1.

To screen for mediators responsible for expanding the CD16^Int^ neutrophil population, we measured 20 cytokines/chemokines in COVID-19 patient plasma samples (Supplemental Table 5). As shown in Supplemental Figure 5B, plasma levels of IL-10, IL-1RA, MCP-1, and MIP-1α positively correlated with the percentage of CD16^Int^ neutrophils but correlated negatively with the percentage of CD16^hi^ neutrophils. No correlations were noted in the CD16^lo^ neutrophil population (data not shown). These 4 cytokines/chemokines are likely to be involved in neutrophil trafficking and migration. Therefore, it remains to be determined whether these factors contribute to the emergence of CD16^Int^ neutrophils in patients with severe COVID-19.

### Contribution of CD16^Int^ LDNs to systemic cytokine production in patients with COVID-19.

**Figure 6:**
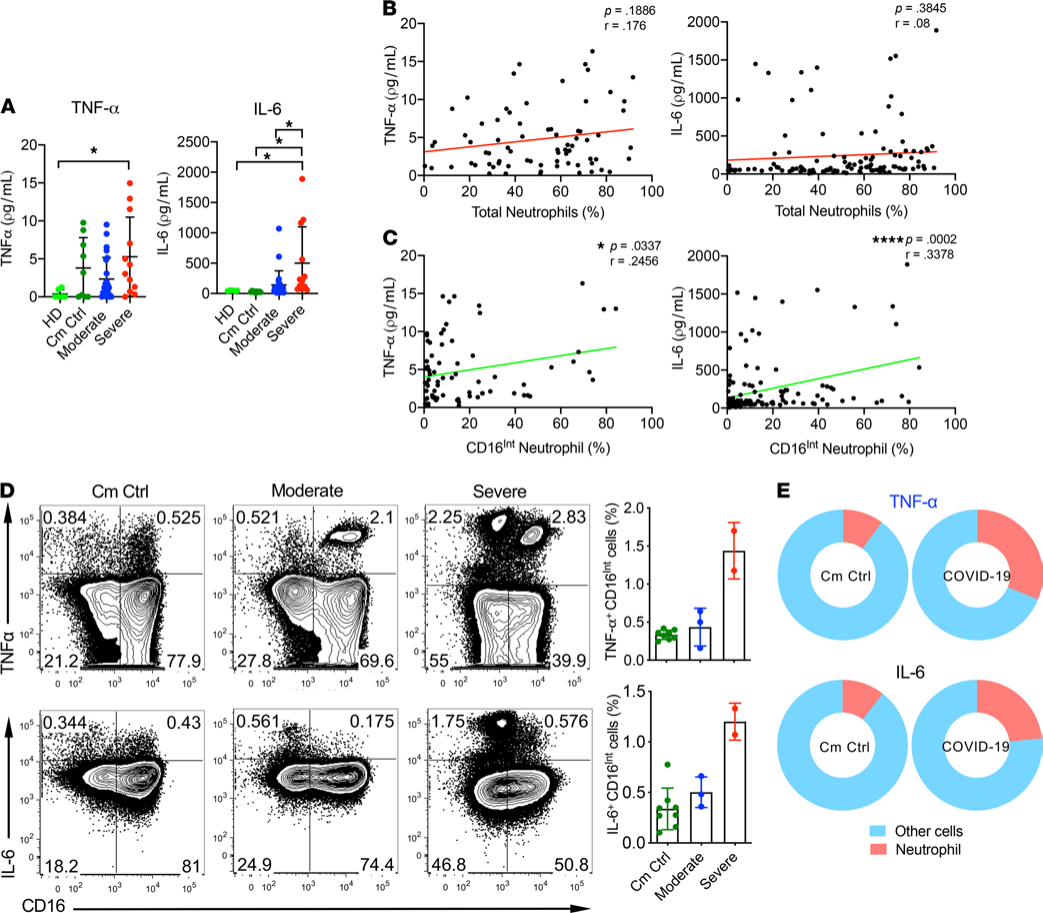
Enhanced cytokine production by CD16^Int^ LDNs in severe COVID-19 patients. (**A**) Plasma concentrations of IL-6 and TNF-α in a single draw from HD (*n* = 6) and Cm Ctrl (*n* = 9) groups and the average value during study enrollment for moderate (*n* = 24) and severe (*n* = 12) COVID-19 patients. (**B** and **C**) IL-6 and TNF-α levels in serial patient draws were then correlated with both the percentage of total neutrophils (**B**) and the percentage of CD16^Int^ neutrophils (**C**) in the corresponding sample as measured by CyTOF. (**D**) Representative dot plots of TNF-α (top) and IL-6 (bottom) production from LPS-stimulated neutrophils cultured from whole blood samples of Cm Ctrl (*n* = 8), moderate patients (*n* = 3), and severe patients (*n* = 2), with accompanying summarized data. (**E**) Pie charts show the relative contribution of neutrophils to the total TNF-α and IL-6 ex vivo pool compared with all other immune cells in Cm Ctrl patients and severe COVID-19 patients. Pearson correlations were used to indicate statistical significance in all correlations. Data are shown as mean ± SD. *P* values were determined using 1-way ANOVA with multiple comparisons. *P < 0.05, ****P < 0.0001.

Patients with severe COVID-19 have elevated levels of proinflammatory cytokines, resulting in cytokine storm (9, 10). Two cytokines found to be consistently elevated among the most severe COVID-19 patients are TNF-α and IL-6 (9, 49). In addition to their effect on innate immunity, both IL-6 and TNF-α activate the extrinsic coagulation cascade by inducing endothelial cell expression of tissue factor (50). Because these activities may contribute to COVID-19 coagulopathy, we determined whether CD16^Int^ LDNs and/or overall neutrophils contributed to the generation of these cytokines and whether they correlated with clinical markers of coagulation and systemic inflammation. Although plasma levels of TNF-α remained low in COVID-19 patients, TNF-α levels were significantly higher in the severe COVID-19 group compared with healthy donors. IL-6 levels in severe COVID-19 patients were significantly increased above those in moderate COVID-19 patients, comorbidity control patients, and healthy donors (Figure 6A). Plasma levels of TNF-α and IL-6 did not significantly correlate with total neutrophil percentage (Figure 6B). The percentage of CD16^Int^ LDNs, however, showed a significant positive correlation with TNF-α and IL-6 levels across all COVID-19 patients (Figure 6C).

Next, we examined whether neutrophils directly contribute to these systemic cytokine pools. CD16^Int^ neutrophils in the severe patients released higher amounts of TNF-α and IL-6 compared with moderate or comorbidity control patients (Figure 6D). Additionally, neutrophils from severe COVID-19 patients accounted for an increased proportion of cytokine-producing cells compared with comorbidity control patients (Figure 6E). TNF-α levels demonstrated a significant correlation with platelet counts and lactate dehydrogenase (LDH) levels but no correlation with D-dimer and ferritin (Supplemental Figure 6A). In contrast, IL-6 levels were positively correlated with the levels of D-dimer, ferritin, and LDH and negatively correlated with platelet count (Supplemental Figure 6B). Overall, these data suggest that neutrophils, particularly the CD16^Int^ LDN subset, are important contributors to the elevated cytokine levels seen in patients with COVID-19.

### Clinical significance of CD16^Int^ neutrophils in patients with COVID-19.

**Figure 7:**
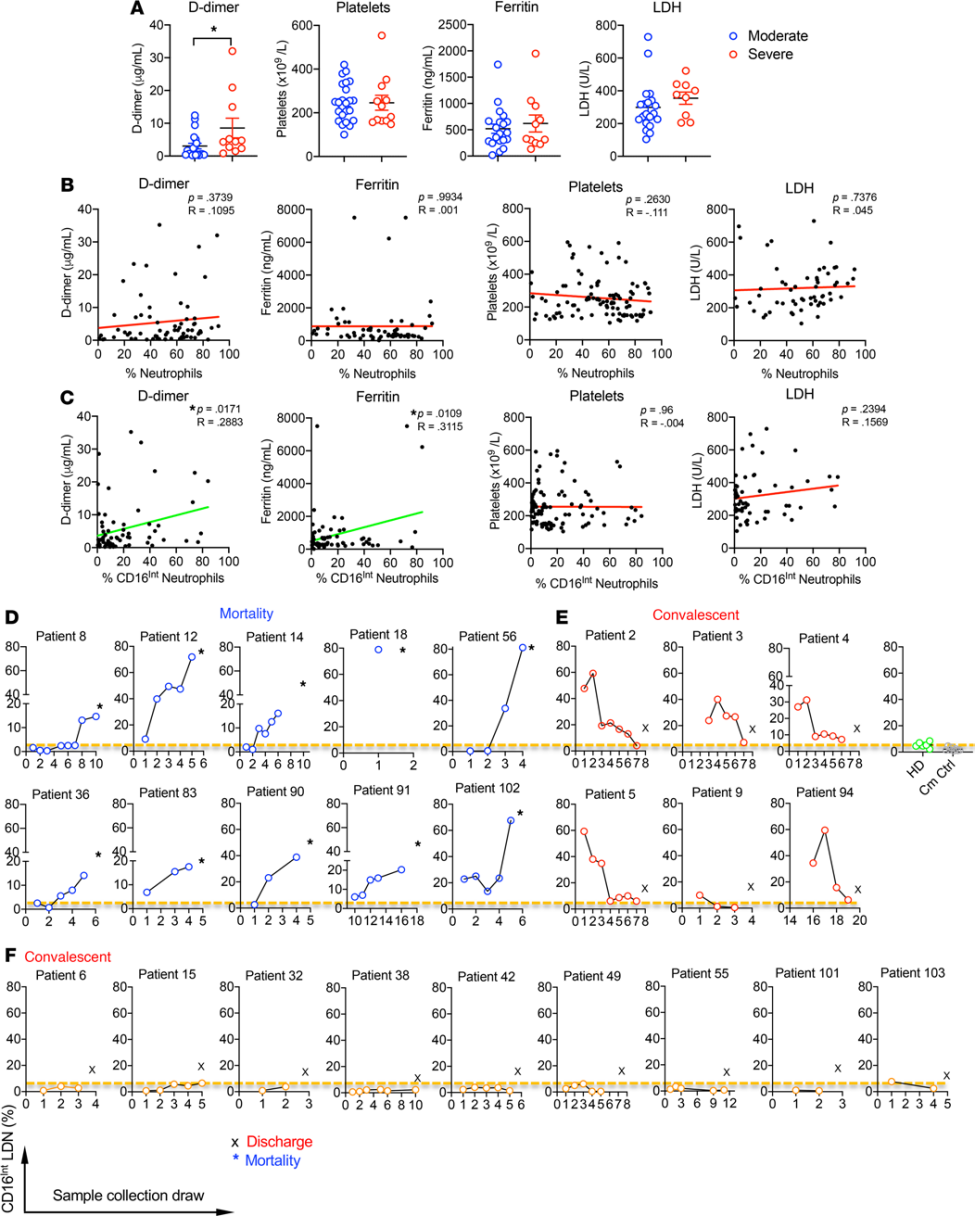
Correlations of clinical coagulation and systemic inflammation indicators and disease outcomes with CD16^Int^ LDNs. (**A**) For severe and moderate patients, the clinical values of D-dimer, ferritin, platelets, and LDH were acquired from patient charts. The average value of serial blood draws from patients was used. An unpaired Student’s *t* test was used to determine significance. *P < 0.05. (**B** and **C**) The D-dimer, ferritin, platelet number, and LDH level for all COVID-19 patient samples were correlated with the total CD66b^+^ neutrophil percentage (**B**) or CD16^Int^ (**C**) neutrophils in the PBMCs. For all correlation data, a line of best fit is shown to visually depict correlation, with a green line representing a statistically significant correlation and a red line representing a nonsignificant correlation. Pearson correlations were used to determine statistical significance in all correlations, where *P < 0.05. (**D**–**F**) Longitudinal, serial blood draws from our patient cohort (25 patients) enabled us to track the CD16^Int^ LDN population percentage in Ficoll-isolated PBMCs over the course of patient hospitalization and correlate it with patient clinical outcomes. (**D**) COVID-19 patients (*n* = 10) with mortality showed an increased CD16^Int^ LDN trend over time. (**E**) COVID-19 patients (*n* = 6) with convalescence showed a decreased CD16^Int^ LDN trend over time and the frequency of CD16^Int^ LDN in the blood draw before discharge was similar to the level in heathy donors (HD, *n* = 6) or comorbidity control patients (Cm Ctrl, *n* = 9). (**F**) COVID-19 patients (*n* = 9) with convalescence showed low levels of CD16^Int^ LDNs similar to those from HD over time. Dotted line represents the average level of CD16^Int^ LDNs in PBMCs from HD (*n* = 6). All data are shown as mean ± SD.

Two clinical markers used to monitor coagulation state are D-dimer and platelet count, where increased D-dimer levels and decreased platelet counts are associated with enhanced coagulation (11). Our severe COVID-19 cohort showed elevated D-dimer levels compared with patients with moderate disease (Figure 7A). Platelet counts were similar between 2 groups. Two clinically relevant markers used to monitor systemic inflammation are ferritin and LDH (51). Ferritin levels were elevated above the normal range in our COVID-19 patients; however, there was no difference between patients with moderate and severe disease. LDH levels were similar in the severe cohort versus the moderate group (Figure 7A).

To determine whether total neutrophil percentage can identify patients with a high risk of thromboembolism, the neutrophil percentage was correlated with D-dimer, ferritin, platelet count, and LDH levels. There was no significant correlation between neutrophil percentage and any of these markers (Figure 7B). In contrast, the CD16^Int^ neutrophil percentage significantly correlated with D-dimer and ferritin levels, whereas there was no correlation with platelet count or LDH level (Figure 7C). Longitudinal analyses of individual patients’ CD16^Int^ neutrophil populations with D-dimer demonstrated a significant relationship and a pronounced phenotype. Supplemental Figure 7A shows a representative severe patient in whom rising D-dimer levels correlated with an increasing CD16^Int^ neutrophil population within their peripheral blood until their death (Supplemental Figure 7A). In contrast, both D-dimer and CD16^Int^ LDN percentage in 1 patient from the moderate group were only marginally elevated throughout the hospital stay until discharge (Supplemental Figure 7B). These findings suggest that the CD16^Int^ neutrophil percentage rather than overall neutrophil percentage correlates with coagulation status and clinical outcome in patients with COVID-19.

Tracking the CD16^Int^ neutrophil population over the course of each patient’s hospital stay revealed an association between clinical outcomes and the percentage of CD16^Int^ neutrophils (Figure 7, D–F). The longitudinal blood samples were collected from 25 patients and the frequency of CD16^Int^ LDNs was monitored over time. In patients who died, the percentage of CD16^Int^ LDNs increased over time and reached the highest level on the last sample obtained before death (Figure 7D). For patients recovering from COVID-19, 2 scenarios were observed. One group of patients showed an initial high percentage of CD16^Int^ LDNs that gradually decreased to basal levels prior to discharge (Figure 7E). A second group of patients showed low percentages of CD16^Int^ LDNs for the duration of the hospitalization until discharge (Figure 7F). Collectively, these findings suggest that an emergence of CD16^Int^ LDNs is common in patients with COVID-19 and that changes in the percentage of CD16^Int^ LDNs predict both improvement and decline in clinical status.

## Discussion

The primary finding of our study is the emergence of a subpopulation of LDNs in patients with COVID-19 that associates with disease severity and changes over time in parallel with changing coagulation and clinical status. Although our severe COVID-19 patients showed an increased neutrophil percentage and increased NLR, neither measurement was associated with coagulation status. We describe the emergence of a unique LDN subpopulation in COVID-19 patients. Previous studies have shown that LDNs are expanded in severe infection and autoimmune disorders such as lupus (37, 52). Indeed, comorbidity COVID-19–negative control patients had significantly increased LDNs within the PBMC population. However, LDNs are a heterogenous population (53) that can be further classified as CD16^hi^, CD16^Int^, and CD16^lo^. Our study showed that CD16^Int^ LDNs are only increased in COVID-19 patients, suggesting that SARS-CoV-2 infection specifically drives expansion of this subset. In addition, severe patients have a greater percentage of CD16^Int^ LDNs than moderate patients, indicating that CD16^Int^ LDNs are correlated with disease severity. Our data expand on the findings of 2 recent studies showing emergence of dysfunctional LDNs in severely ill COVID-19 patients (47, 54).

LDNs are classically considered to be immature neutrophils (55), and our CD16^Int^ LDN population showed a band-shaped nucleus, resembling immature neutrophil morphology. Although previous studies suggested the emerging neutrophils are immature with phenotypic signs of immunosuppression and dysfunction (47, 54), our RNA-Seq data revealed that the CD16^Int^ LDNs had a potent proinflammatory gene signature and demonstrated increased neutrophil degranulation, NET formation, and phagocytosis. NET formation has been reported in severe COVID-19 pulmonary autopsies (27, 28). Serum levels of cell-free DNA, DNA-MPO complexes, and citrullinated histone H3 are increased in COVID-19 patients (56), further supporting the notion that NETs play a critical role in lung immunopathogenesis in severe COVID-19 patients (57). In addition to expression of NET-related genes, we observed that CD16^Int^ LDNs spontaneously formed large numbers of NETs. Collectively, our findings indicate that CD16^Int^ LDNs are morphologically immature but functionally competent with a hyperactivated phenotype.

Evidence suggests that neutrophils aggregate with platelets in COVID-19, leading to microvascular thrombosis and subsequent lung damage (28). Our data showed that neutrophil-platelet aggregates contained CD16^hi^ and CD16^Int^ neutrophils; however, a higher percentage of platelets with activation markers were present in the CD16^Int^ neutrophil aggregates. This is consistent with RNA-Seq data showing genes related to platelet activation and degranulation were enriched in CD16^Int^ LDNs. Additionally, CD40 expression was higher in these aggregates, and the frequency of CD40^+^CD16^Int^ LDNs highly correlated with D-dimer levels in patients with COVID-19. Although it is possible that platelet activation could activate neutrophils, a recent study suggests that the activation status of neutrophils is more important than platelet activation in COVID-19–related thrombosis (58). Overall, our results suggest that CD16^Int^ neutrophils may be capable of promoting coagulation and thrombosis and could play a prominent role in CAC, though future studies are needed to show a direct connection between CD16^Int^ neutrophils and the formation of platelet aggregates.

Neutrophil infiltration of the lung is accompanied by lung edema, endothelial injury, and epithelial injury, which are hallmark events in the development of ARDS (29, 59). Our finding that neutrophils were the major immune cells in the BAL fluid from severe COVID-19 patients is consistent with previous reports (44, 60). In the 6 patients analyzed, we showed that the CD16^Int^ neutrophil subpopulation consistently constituted more than 60% of neutrophils in the BAL fluid. Those CD16^Int^ BAL fluid neutrophils expressed CXCR3 but lost CD44 and CD38 expression compared with CD16^Int^ neutrophils in the blood. In addition, CD16^Int^ BAL fluid neutrophils expressed higher levels of CXCR3 than the CD16^hi^ population. The elevated potent neutrophil chemoattractants, including the CXCR3 ligand IP-10 (CXCL10), in the BAL fluid may preferentially recruit CXCR3^+^CD16^Int^ neutrophils into alveoli and BAL fluid. The mechanism by which CD16^Int^ neutrophils recruited to the lungs lose CD44 and CD38 expression is unknown; however, neutrophils undergoing transmigration from the vasculature undergo a number of phenotypic changes, including release of proteolytic enzymes (61, 62). The downregulation of CD44 may enhance trafficking of these cells into the lung, given that previous studies report that CD44-deficient mice show markedly increased migration of neutrophils into the lungs after induction of bacterial pneumonia or hypoxia-induced injury (41, 63, 64). Strikingly, CD16^Int^ neutrophils from BAL fluid completely lose CD38 expression. CD38 was reported to play a role in neutrophil chemotaxis to bacterial formylated peptide chemoattractants (46). Our results suggest the hypothesis that reduced CD38 expression may inhibit CD16^Int^ neutrophil chemotaxis, thereby limiting emigration from the lung. BAL fluid also demonstrated markedly increased levels of TNF-α and IL-6. Our data showed that CD16^Int^ neutrophils were capable of producing increased levels of these cytokines compared with comorbidity controls. Hence, the recruitment of CD16^Int^ neutrophils to the lung in COVID-19 may also play an important role in cytokine production, leading to the development of ARDS observed in the most severely ill COVID-19 patients.

To address the question of which mediators are responsible for expanding the CD16^Int^ neutrophil population, we measured the levels of cytokines/chemokines in COVID-19 patient plasma samples. The plasma levels of IL-10, IL-1RA, MCP-1, and MIP-1α positively correlated with the percentage of CD16^Int^ neutrophils and negatively correlated with the percentage of CD16^hi^ neutrophils. Interestingly, a recent study reported that IL-10 and IL-1RA levels are associated with disease severity in COVID-19 patients using longitudinal blood samples (65). In addition, a previous report also showed that ICU patients had higher plasma levels of MCP-1 and MIP-1α (9). Collectively, these correlation studies further support our conclusion that CD16^Int^ neutrophils play a critical role in disease development and progression. Although the levels of these 4 cytokines/chemokines significantly correlate with percentages of CD16^Int^ neutrophils, it is currently unknown whether these cytokines actually stimulate expansion of CD16^Int^ neutrophils in patients with severe COVID-19.

Recent publications promoted the use of antiinflammatory agents in the treatment of COVID-19 (66). Numerous case reports suggest that COVID-19 patients with a history of inflammatory autoimmune diseases like rheumatoid arthritis or inflammatory bowel disease have a milder course of infection (67, 68). In the context of the data presented here, the reduced disease severity in autoimmune diseases could be due to drug-induced neutropenia or to decreased TNF-α/IL-6 levels from antibody treatment. Hesitation to use cytokine-blocking antibodies like tocilizumab, adalimumab, and etanercept exists because of concerns that restraining immune function will promote the viral infection (69). The results with dexamethasone treatment, however, have shifted opinion toward acceptance of immune modulation and suppression as successful treatment (70). However, the challenge to correctly identify patients who could benefit from immunosuppressive regimens like dexamethasone or anti–IL-6 therapy remains. Based on the data we present here, we propose that CD16^Int^ LDN levels could serve as a predictor of risk for progressive ARDS and CAC, thereby identifying patients in whom implementation of antiinflammatory therapy may be beneficial.

## Methods

### Study participants and clinical data.

Inclusion criteria were all hospitalized adults (older than 18) who had positive COVID-19 results and consented to this study. Exclusion criteria included age younger than 18 and refusal to participate. COVID-19 patients enrolled in this study were diagnosed with a 2019-CoV detection kit using real-time reverse-transcriptase PCR performed at the University of Louisville hospital laboratory from nasal pharyngeal swab samples obtained from patients. The grouping of COVID-19 patients into moderate group versus severe group was based on the initial clinical presentation at the time of enrollment. Severe group participants were patients with confirmed COVID-19 who required mechanical ventilation, and this group had blood drawn daily along with their standard laboratory work. Moderate group participants were patients with confirmed COVID-19 who were hospitalized without mechanical ventilation and had blood drawn every 2 to 3 days along with their standard laboratory work. All patients with COVID-19 were followed by the research team daily, and the clinical team was blinded to findings of the research analysis to avoid potential bias.

The demographic characteristics (age, sex, height, weight, BMI, and clinical data: symptoms, comorbidities, laboratory findings, treatments, complications, and outcomes) were collected prospectively. All data were independently reviewed and entered into the computer database. For hospital laboratory CBC tests, normal values are the following: white blood cell (4.1–10.8 × 10^3^/μL), hemoglobin (13.7–17.5 g/dL), platelet (140–370 × 10^3^/μL). For hospital laboratory inflammatory and coagulation markers, normal values are the following: D-dimer (0.19–0.74 μg/mL FEU), ferritin (7–350 ng/mL), LDH (100–242 U/L).

### Plasma and PBMC isolation.

Whole blood samples were centrifuged at 541*g* for 10 minutes. Plasma was aspirated and aliquoted into 1 mL Eppendorf tubes and immediately stored at –80°C until future use. The remaining cell layers were diluted with an equal volume of complete RPMI 1640. The blood suspension was layered over 5 mL of Ficoll-Paque (Cedarlane Labs) in a 15 mL conical tube. Samples were then centrifuged at 845*g* for 30 minutes at room temperature (RT) without brake. The mononuclear cell layer was then transferred to new 15 mL conical tubes and washed with complete RPMI 1640. The cell pellet was resuspended in 3 mL of RPMI 1640 and counted for sample processing.

### Whole blood analysis.

For whole blood analysis, 150 μL of whole blood was lysed with 2 mL of ACK buffer for 10 minutes. Cells were spun down and washed once with PBS. Cells were then stained with viability dye–APC-Cy7, CD45-PeCy7, CD66b-PE, and CD-16 APC (BioLegend) for 30 minutes at 4°C prior to washing and analysis of a FACSCanto (BD Biosciences).

### CyTOF mass cytometry sample preparation.

Mass cytometry antibodies (Supplemental Table 6) were either purchased preconjugated (Fluidigm) or were conjugated in house using MaxPar X8 polymer kits or MCP9 polymer kits (Fluidigm) according to the manufacturer’s instructions. PBMCs were isolated as described above. The samples were stained for viability with 5 μM cisplatin (Fluidigm) in serum-free RPMI 1640 for 5 minutes at RT. The cells were washed with complete RPMI 1640 for 5 minutes and stained with the complete antibody panel for 30 minutes at RT. Cells were then washed and fixed in 1.6% formaldehyde for 10 minutes at RT, then incubated overnight in 125 nM of Intercalator-Ir (Fluidigm) at 4°C.

### CyTOF data acquisition.

Prior to acquisition, samples were washed twice with cell staining buffer (Fluidigm) and kept on ice until acquisition. Cells were then resuspended at a concentration of 1 million cells/mL in cell acquisition solution containing a 1:9 dilution of EQ 4 Element Beads (Fluidigm). The samples were acquired on a Helios (Fluidigm) at an event rate of less than 500 events/s. After acquisition, the data were normalized using bead-based normalization in the CyTOF software. The data were gated to exclude residual normalization beads, debris, dead cells, and doublets, leaving DNA^+^CD45^+^Cisplatin^lo^ events for subsequent clustering and high-dimensional analyses.

### CyTOF data analysis.

CyTOF data were analyzed using a combination of the Cytobank software package (71) and the CyTOF workflow (72), which consists of a suite of packages (73–77) available in R (https://www.r-project.org/). For analysis conducted within the CyTOF workflow, FlowJo Workspace files were imported and parsed using functions within flowWorkspace (73) and CytoML (74). An arcsinh transformation (cofactor = 5) was applied to the data using the dataPrep function within CATALYST (75) and stored as a “singlecellexperiment” object (76). Cell population clustering and visualization were conducted using FlowSOM (78) and ConsensusClusterPlus (77) within the CyTOF workflow and using the viSNE application within Cytobank. Clustering was performed using data across all donors and time points. Additionally, clustering was performed either using all live CD45^+^ cells or after gating on CD66b^+^ neutrophils.

### Wright-Giemsa stain.

Five hundred thousand PBMCs were stained with viability dye–APC-Cy7, CD45-PerCP-Cy5.5, CD66b-PE, and CD16-APC for 30 minutes at 4°C. Cells were then sorted based on CD16 expression using a BD Biosciences FACSAria III. After collection, cells were spun down at 541*g* for 8 minutes. Cells were resuspended in 200 μL and spun onto a microscope slide using a Shandon CytoSpin3 (Thermo Fisher Scientific). Slides were then air-dried for 10 minutes prior to staining. For the Wright-Giemsa stain (Shandon Wright-Giemsa Stain Kit, Thermo Fisher Scientific), slides were dipped in Wright-Giemsa stain solution for 1 minute and 20 seconds. After blotting off excess stain, slides were dipped in Wright-Giemsa buffer for 1 minute and 20 seconds. Slides were blotted to remove excess buffer. Slides were then dipped into the Wright-Giemsa rinse solution for 10 seconds using quick dips. The backs of the slides were wiped and set to dry in a vertical position for 10 minutes prior to analysis on an Aperio Scan Scope (Leica Biosystems).

### RNA extraction and sequencing.

PBMCs from severe COVID-19 patients were washed and stained with viability dye–APC-Cy7, CD45-PerCP, CD66b-PE, CD16-APC for 30 minutes at 4°C. CD16^hi^ and CD16^Int^ CD66b^+^ neutrophils were sorted by a BD Biosciences FACSAria III. Cells were then lysed in TRIzol (Thermo Fisher Scientific), and RNAs were extracted with a QIAGEN RNeasy kit. Libraries were prepared using the Universal Plus mRNA-Seq with NuQuant (NuGen). Sequencing was performed on the University of Louisville Brown Cancer Center Genomics Core Illumina NextSeq 500 using the NextSeq 500/550 75 cycle high output kit v2.5. The RNA-Seq data have been deposited into NCBI’s Gene Expression Omnibus database with the accession number GSE154311.

### Phagocytosis assay.

Cells were acquired from whole blood after ACK lysis. The pHrodo Green *S*. *aureus* BioParticles phagocytosis kit (Thermo Fisher Scientific) was used, where 100 μL of the reconstituted particles were added to the cell suspension and incubated for 1 hour at 37°C. Samples were lightly mixed every 20 minutes. The reaction was stopped with 1 mL of cold PBS. Cells were then stained for viability, CD45, CD66b, and CD16 (BioLegend). Samples were acquired by BD FACSCanto.

### NET assay.

NET formation was tested using confocal microscopy as previously described (79). Sorted CD16^Int^ (0.5 × 10^6^ cells/well) were resuspended in NET media (colorless RPMI + 0.5% BSA +10 mM HEPES) and seeded onto sterile acid–washed coverslip coated with (1 mg/mL) poly-l-lysine. Cells were incubated for 60 minutes in CO_2_ incubator. After incubation, cells were fixed with 2% PFA for 30 minutes, washed twice with PBS, and blocked with 1% BSA in PBS for 1 hour. NETs were determined by extracellular colocalization of anti-human lactoferrin antibody (1:500 dilution, MP Biomedicals, catalog 55040) with DAPI (600 nM for 10 minutes) nuclear stain. The secondary antibody utilized was Alexa Fluor 647 (1:1000; Invitrogen, Thermo Fisher Scientific, catalog A21246). Confocal images and *Z*-stacks (1 μm thickness for each slice) were obtained by the Fluoview FV1000 confocal microscope (Olympus) with the 63× oil objective. Confocal *Z*-stack images were used to quantify colocalization of extracellular DNA and lactoferrin using IMARIS v9.6 software (Oxford Instruments).

### Neutrophil-platelet aggregates.

Whole blood samples from COVID-19 patients were diluted with Tyrode’s/HEPES buffer at 1:5. Cells were stained with anti–human CD66b (clone G10F5, BioLegend), CD16 (clone 3G8, BioLegend), and CD40 (clone 5C3, BioLegend); platelet marker anti–human CD41 (clone HIP8, BioLegend); and platelet activation marker anti–human CD62P (clone AK4, BioLegend) for 10 minutes at RT in the dark. Cells were fixed with 1% paraformaldehyde for 10 minutes and then acquired by FACSCanto.

### BAL fluid collection.

Nonbronchoscopic protected BAL was performed using a closed suction system with a 14 F 40 cm catheter inside to prevent aerosolization. After injection of 30–40 mL sterile normal saline into the endotracheal tube, the suction catheter was inserted through the endotracheal tube and blindly advanced into the distal airways until resistance was felt. The catheter was wedged in that position, and aspirate was collected in a sterile container into a sputum trap cup. The procedure was repeated if the aspirated fluid was less than 5 mL.

### U-PLEX assays.

U-PLEX Viral Combo 1 (human) kit, which includes 20 analytes, was purchased from Meso Scale Diagnostics. The plate was read with a MESO QuickPlex SQ 120 imager and analyzed using Discovery Workbench v4.0 software. The assay was performed according to the manufacturer’s instructions.

### TNF-α and IL-6 quantification.

Plasma concentrations of TNF-α and IL-6 were measured using ELISA kits (BioLegend). The operating procedure provided by the manufacturer was followed. For each sample, 100 μL of plasma was used. The OD at 450 nm was measured using a Synergy HT microplate reader (BioTek). Concentrations of TNF-α and IL-6 were determined using the standard curves. A few OD readings fell outside of the range of the standard curve, in which case a line of best fit was used to extrapolate the data.

### Ex vivo neutrophil stimulation.

Whole blood (150 μL) was lysed with ACK buffer. One million cells were seeded in a 24-well plate and cultured with Brefeldin A solution for 20 minutes at 37°C. Cells were then stimulated with 250 ng/mL of LPS for 10 hours at 37°C. After stimulation, cells were collected and washed with PBS prior to cell surface staining with viability dye–APC-Cy7, CD45-PE-Cy7, CD66b-PE, and CD16-APC for 30 minutes at 4°C. Cells were washed again with PBS before fixation (BioLegend intracellular fixation buffer) for 30 minutes at RT. Cells were washed twice with permeabilization buffer (BioLegend perm wash buffer). Cells were incubated with TNF-α–PerCP-Cy5.5 and IL-6–FITC overnight prior to washing and analysis on BD FACSCanto.

### Statistics.

The 2-tailed, unpaired Student’s *t* test was used to determine the significance of differences between 2 groups. One-way ANOVA was used to determine differences between multiple groups. Since we had a varied number of observations for each patient, we applied linear mixed-effect models along with Wald’s test statistics to compare the group differences (80), where the group was considered as fixed effects and patients were considered random effects. To examine the association between 2 variables, we estimated the marginal Pearson correlation coefficient and tested its significance (81). The marginal Pearson correlation coefficient captures the association between 2 variables at the population level. The analyses were carried out in the statistical software R and GraphPad Prism, Version 9.0.0. A statistical test was claimed significant if *P* was less than 0.05.

### Study approval.

The IRB at University of Louisville approved the present study, and written informed consent was obtained from either participants or their legal authorized representatives (IRB 20. 0321).

## Author contributions

SMM, JH, and JY initiated this study. SMM, AEG, and XH led data collection and analysis. CTW and DT performed data analysis for clustering algorithms. ECR performed RNA-Seq analysis. CKK and SMU performed the NET assay. JY, JH, CD, HZ, LC, SMU, and KRM contributed to experimental design. EAC, RC, OC, SEB, SPC, ZCM, JC, and JH recruited patients and obtained their consent. JH and MRW performed patient chart review. SG and SSB contributed to the U-PLEX assay. MK performed and supervised all biostatistical analysis. SMM, AEG, and JY wrote the manuscript. CTW, LC, MK, SMU, KRM, and JH edited and revised the manuscript. JH and JY designed the study, implemented the experiments, and supervised the data analysis. SMM and AEG share co–first author position. The method used in assigning the authorship order among these authors is based on the time order in which they worked on this study.

## Supplementary Material

Supplemental data

## Version 1 05/10/2021

Electronic publication
