# Supplementary material for: A specific low-density neutrophil population correlates with hypercoagulation and disease severity in hospitalized COVID-19 patients
Source: JCI Insight. 2021 May 10;6(9):e148435. doi: 10.1172/jci.insight.148435 (PMC8262329; doi:10.1172/jci.insight.148435)

Inventory for Supplemental materials

1. Supplemental Tables 1-6

2. 7 Supplemental Figures

Variables	Healthy Control (N=6)	Comorbid Healthy Control (N=9)	COVID-19 (N=53)
**Sex – no (%)**			
Male	4 (66.6)	5 (55.5)	24 (42.3)
Female	2 (33.3)	4 (44.4)	28 (57.7)
**Age - year**			
Mean ± SD	49.2 (15.1)	65.9 (16.7)	60.8 (17.75)
Median (IQR)	53.5 (24.25)	63 (24)	63 (28.25)
Range	28-68	41-93	28-95
**BMI**			
Mean ± SD	28.75 (3.5)	30.82 (13.1)	31.83 (9.07)
Median (IQR)	29.55 (3.13)	29.15 (11.22)	30.6 (14.48)
Range	16.3-27	16.4 - 60.2	17.7- 51.8
**Ethnicity – no (%)**			
AA	0 (0)	2 (22)	18 (34.6)
White	6 (100)	7 (78)	33 (63.4)
Hispanic	0 (0)	0 (0)	1 (2)
**Time from symptoms to hospital admission, days**			
Mean ± SD			5.17 (5.41)
Median (IQR)			4 (4.75)
Range			0-14
**Comorbidity – no (%)**			
Mean ± SD	1 (.81)	6 (2.62)	6.1 (3.7)
Median (IQR)	1 (1.5)	7 (4)	4
Range	0-2	1-9	1-18
hypertension	2 (33.3)	7 (77.7)	39 (75)
Diabetes	0	4 (44.4)	27 (52)
Respiratory System	0	6 (66.6)	21 (40.4)
Cardiovascular Disease	0	4 (44.4)	22 (42.3)
Kidney Disease	0	1 (11.1)	11 (21.15)
**Treatment**			
Hydroxychloroquine – no (%)			8 (15.38)
Convalescent Plasma – no (%)			8 (15.38)
Dexamethasone – no (%)			12 (23.1)
Remdesivir – no%			11 (21.15)

Table S1: Total study participant demographics and clinical information

Neutrophil phenotyping	Healthy Control (N=6)	Comorbid Healthy Control (N=9)	COVID-19 Moderate (N=24)	COVID-19 Severe (N=12)
**Sex – no (%)**				
Male	4 (66.6%)	5 (55.5%)	13 (54%)	6 (50%)
Female	2 (33.3%)	4 (44.4%)	11 (46%)	6 (50%)
**Age – year**				
Mean ± SD	49.2 (15.1)	65.9 (16.7)	63.1 (18.55)	63.6 (15.1)
Range	28-68	41-93	28-95	28-84
**BMI**				
Mean ± SD	28.75 (3.5)	30.82 (13.1)	31.15 (7.67)	36.4 (9.86)
Range	16.3-27	16.4 - 60.2	17.7- 48.8	21.2-49.8
**Ethnicity – no (%)**				
AA	0 (0)	2 (22)	9 (37.5)	7 (58.3)
White	6 (100)	7 (78)	15 (62.5)	5 (41.6)
**Comorbidity – no (%)**				
Mean ± SD	1 (.81)	6 (2.62)	4.58 (3.21)	4.7 (3.17)
Range	0-2	1-9	1-12	1-12

Table S2: Neutrophil phenotyping study participant demographics and clinical information

Platelet study	COVID-19 Patients
**Sex – (Total, n=13)**	
Male	7 (53.8%)
Female	6 (46.2%)
**Age - year**	
Mean ± SD	52.15 ± (20.21)
Range	21- 84
**BMI**	
Mean ± SD	32.4 ± (8.8)
Range	21.1 – 45.9
**Ethnicity**	
White	11(84.6%)
African American	2 (15.4%)
**Comorbidities**	
Mean ± SD	6.23 ± (4.16)
Range	1-18

Table S3. Platelet study participant demographics and clinical information

BAL study	COVID-19 Patients
**Sex – (Total, n=6)**	
Male	2 (33%)
Female	4 (66%)
**Age - year**	
Mean ± SD	63.67 ± (14.06)
Range	40-83
**BMI**	
Mean ± SD	35.36 ± (8.65)
Range	22.2 - 45.8
**Ethnicity**	
White	4(66%)
African American	2 (33%)
**Comorbidities**	
Mean ± SD	5 ± (3)
Range	0-9

Table S4. BAL fluid study participant demographics and clinical information

Plasma	COVID-19 Patients
**Sex – (Total, n=36)**	
Male	14 (39%)
Female	22 (61%)
**Age - year**	
Mean ± SD	63.3 ± (17.09)
Range	28-95
**BMI**	
Mean ± SD	31.51 ± (8.9)
Range	17.7 – 49.8
**Ethnicity**	
White	21(58.3%)
African American	14 (38.9%)
Hispanic	1 (2.7%)
**Comorbidities**	
Mean ± SD	5.5 ± (6.04)
Range	1-18

Table S5. Plasma cytokine/chemokine study participant demographics and clinical information

Table S6. Mass cytometry antibody panel

Antigen	Symbol and Mass	Antibody clone	Source
CD45	89Y	HI30	Fluidigm
CD8	106Cd	RPA-T8	Biolegend-Custom
CD14	110Cd	M5E2	Biolegend- Custom
CD4	111Cd	RPA-T4	Biolegend-Custom
CD11b	112Cd	IRCF44	Biolegend-Custom
CD3	113Cd	UCHT1	Biolegend-Custom
CD20	114Cd	2H7	Biolegend-Custom
CD19	116Cd	HIB19	Biolegend-Custom
CD196	141Pr	G034E4	Fluidigm
CD40	142Nd	5C3	Fluidigm
CD123	143Nd	6H6	Fluidigm
CD69	144Nd	FN50	Fluidigm
CD163	145Nd	GHI/61	Fluidigm
IgD	146Nd	IA6-2	Fluidigm
CD11c	147Sm	Bu15	Fluidigm
CD66b	148Nd	G10F5	Biolegend-Custom
CD45RO	149Sm	UCHL1	Fluidigm
LAG-3	150Nd	11C3C65	Fluidigm
LAMP1	151Eu	H4A3	Fluidigm
CD21	152Sm	BL13	Fluidigm
γδTCR	153Eu	B1	Biolegend-Custom
TIM-3	154Sm	F38-2E2	Fluidigm
CD56	155Gd	HCD56	Fluidigm
CD86	156Gd	IT2.2	Fluidigm
TLR4	158Gd	HTA125	Fluidigm
CD197	159Tb	G043H7	Fluidigm
CD28	160Gd	CD28.2	Fluidigm
CD80	161Dy	2D10.4	Fluidigm
CD79b	162Dy	CB3-1	Fluidigm
CXCR3	163Dy	G025H7	Fluidigm
CXCR5	164Dy	RF8B2	Fluidigm
CD45RA	165Ho	HI100	Biolegend-Custom
CD44	166Er	BJ18	Fluidigm
CD27	167Er	L128	Fluidigm
CD40L	168Er	24-31	Fluidigm
CD25	169Tm	2A3	Fluidigm
CTLA-4	170Er	14D3	Fluidigm
CD68	171Yb	Y1/82A	Fluidigm

CD38	172Yb	HIT2	Fluidigm
HLA-Dr	173Yb	L243	Fluidigm
CD279	174Yb	EH12.2H7	Fluidigm
CD274	175Lu	29E.2A3	Fluidigm
CD127	176Yb	A019D5	Fluidigm
CD16	209Bi	3G8	Fluidigm

Supplemental Figure legends:

Figure S1. COVID-19 patients have increased neutrophils and neutrophil to lymphocyte ratio (NLR). (A) Neutrophil and lymphocyte percentages and the NLR in whole blood as measured by a clinical complete blood count (CBC) in healthy donors (HD), comorbidity control patients (Cm Ctrl), and patients with moderate and severe COVID-19. Data points represent a single time point collected from 6 HDs, 9 Cm Ctrl, and the average values of serial blood samples collected during patient hospitalization from 24 moderate patients and 12 severe patients starting from the day of enrollment. Pie charts depict representative data of the NLR in HDs, severe and moderate patients. **(B)** Representative viSNE plots generated using CytoBank showing decreased CD3 (left), CD4 (middle), and CD8 (right) expression in Cm Ctrl patients, moderate and severe COVID-19 patients as compared to HDs in the $CD45^+$ compartment of PBMCs. Data are presented as mean±SD. p values were determined using a one-way ANOVA with multiple comparisons. *$p< 0.05$, **$p<0.01$, ***$p < 0.001$.

Figure S2. Cluster analysis of neutrophils within the $CD45^+$ PBMCs in HD, Cm Ctrl, and moderate and severe COVID-19 patients. Heatmap of differential expression pattern of lineage and surface markers of neutrophils in PBMCs. The color key identifies the cluster populations.

Figure S3. Differential expression of neutrophil clusters in patients over their clinical course of disease. (A) viSNE plots representing the total $CD66b^+$ neutrophil pool in 4 patients who experienced different clinical courses from days 1, 3 and 5 of study enrollment. Data represent a patient who was classified as severe on days 1, 3 and 5 (top), a patient whose

condition improved, and was transitioned to a moderate patient by day 5 (2nd from top), a patient who remained in the moderate group for the entirely of the study (2nd from bottom), and one patient who progressed from the moderate to severe group (bottom). The dynamic nature of $CD66b^+$ neutrophil populations over the course of disease are highlighted by the black and red circles, where cluster surface marker phenotypes are indicated in S4b. **(B)** Heatmap showing differential surface marker expression on the $CD66b^+$ neutrophil pool, which indicates specific subsets of neutrophil populations within the neutrophil compartment.

Figure S4. Differentially expressed genes and enriched pathways between $CD16^{High}$ and $CD16^{Int}$ LDN from severe COVID-19 patients. (A) Principal component analysis (PCA) by the first two principal components (PC1: 68%; PC2: 18%). $CD16^{High}$ and $CD16^{Int}$ LDN were sorted from three severe COVID-19 patients. Normal density neutrophils (NDN) were obtained from three HDs. The three sample groups segregate from each other with a high aggregation between replicates. **(B)** Heatmaps show differentially expressed genes for GO: neutrophil activation (left) and GO: neutrophil involved immune response (right) between $CD16^{High}$ and $CD16^{Int}$ LDN. **(C)** GSEA analysis shows significant enriched pathways in $CD16^{Int}$ LDN compared to $CD16^{High}$ LDN.

Figure S5. Correlation of plasma levels of cytokine/chemokine with the frequency of $CD16^{Int}$ LDN in the PBMC population. (A) CXCR3 and CD44 expression levels on $CD16^{Int}$ and $CD16^{High}$ neutrophils in BAL fluid samples. Data are shown as mean$\pm$SD. p values were determined using a Student's t-test *p<.05, **p<0.01. **(B)** Plasma IL-10, IL-1RA, MCP-1 and MIP-1α levels in serial patient draws were correlated with both the percent of $CD16^{High}$ and $CD16^{Int}$ neutrophils in the corresponding sample as measured by CyTOF. Pearson correlations

were used to indicate statistical significance in all correlations, where ns= $p \geq .05$, * $p < 0.05$, ** $p<0.01$, *** $p < 0.001$.

Figure S6. Correlation of TNF-a and IL-6 with clinical markers (A) TNF-α plasma concentrations were correlated with the clinically measured values from the same day that the sample was acquired. Samples that fell below the level of detection of the TNF-α ELISA were excluded from correlation data. (**B)** IL-6 plasma concentrations were correlated with the clinically measured D-dimer, ferritin, platelets, and LDH levels from the same day that the sample was acquired. Samples that fell below the level of detection of the IL-6 ELISA were excluded from correlation data. For all correlation data, a line of best fit is shown to visually examine correlation, with a green line representing a statistically significant correlation, a red line representing a non-significant correlation. Pearson correlations were used to determine significance. * $p< 0.05$, ** $p<0.01$, *** $p < 0.001$.

Figure S7. Association of circulating CD16^{Int} neutrophil population with clinical D-dimer levels. Sequential whole blood analysis of the CD16^{Int} neutrophil population (middle circle) for severe (**A**) and moderate (**B**) COVID-19 patients overlaid with clinical D-dimer quants from the corresponding days.

Morrissey et al, Supplemental Figure 1

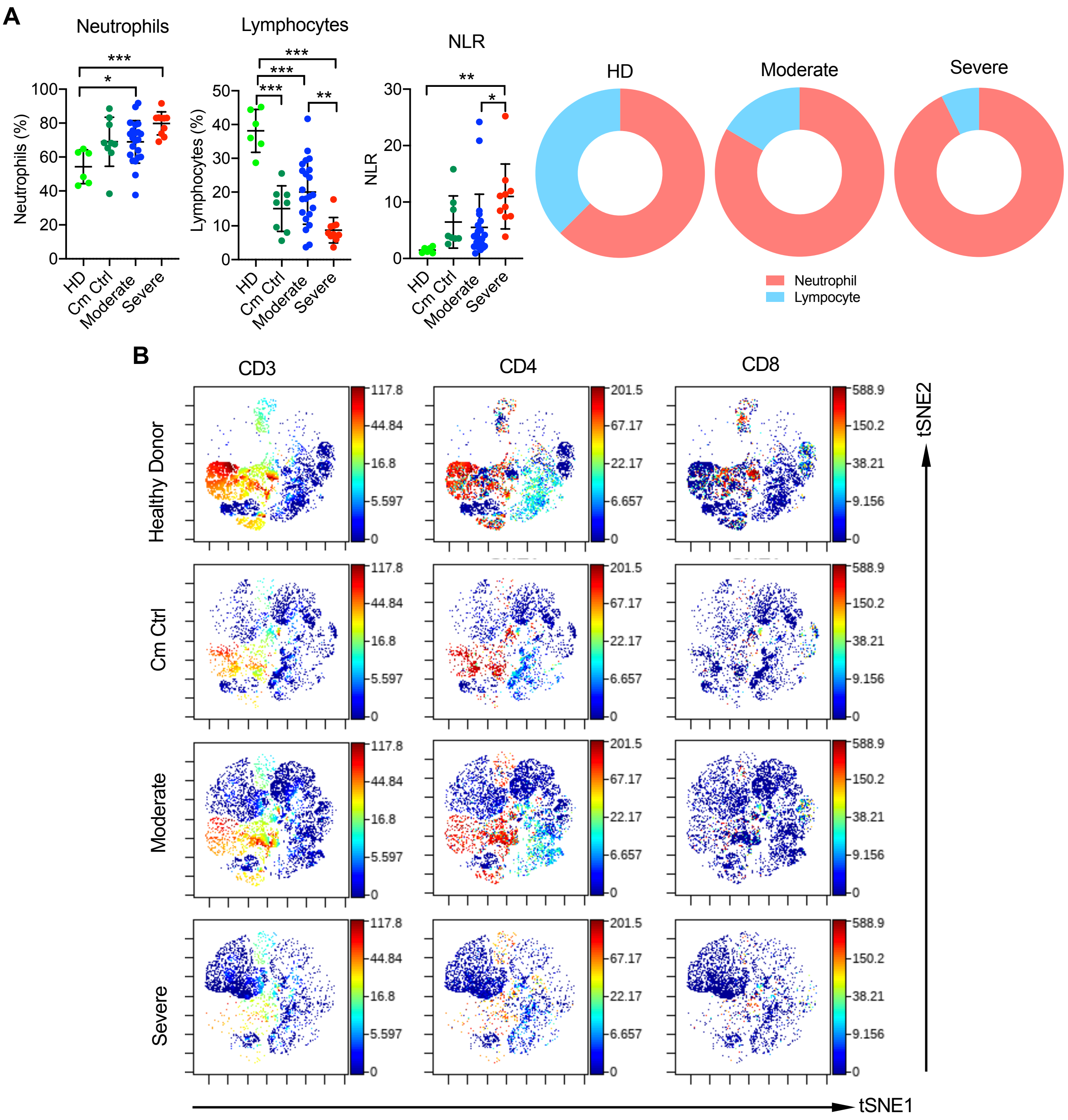

Morrissey et al, Supplemental Figure 2

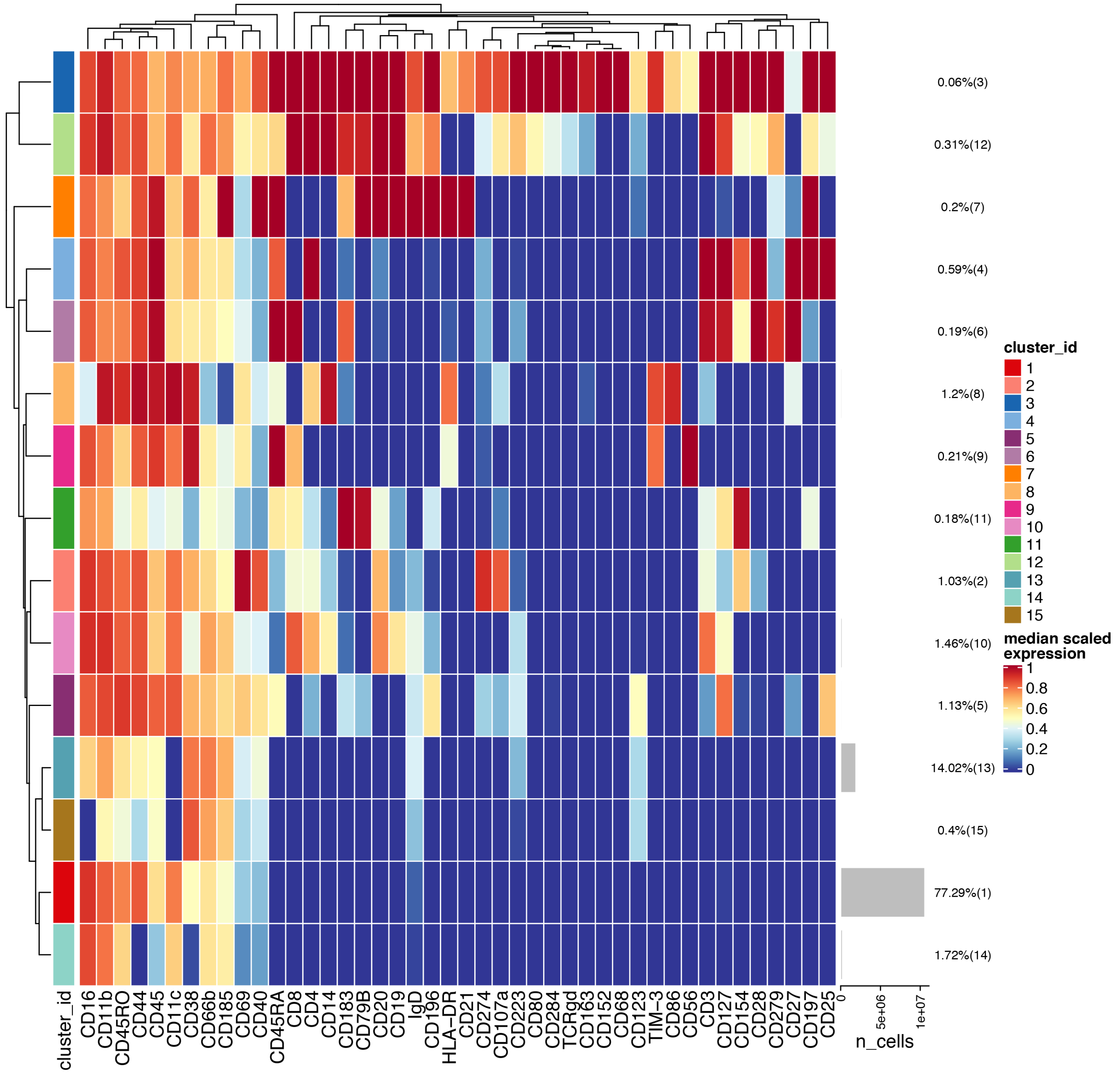

Morrissey et al, Supplemental Figure 3

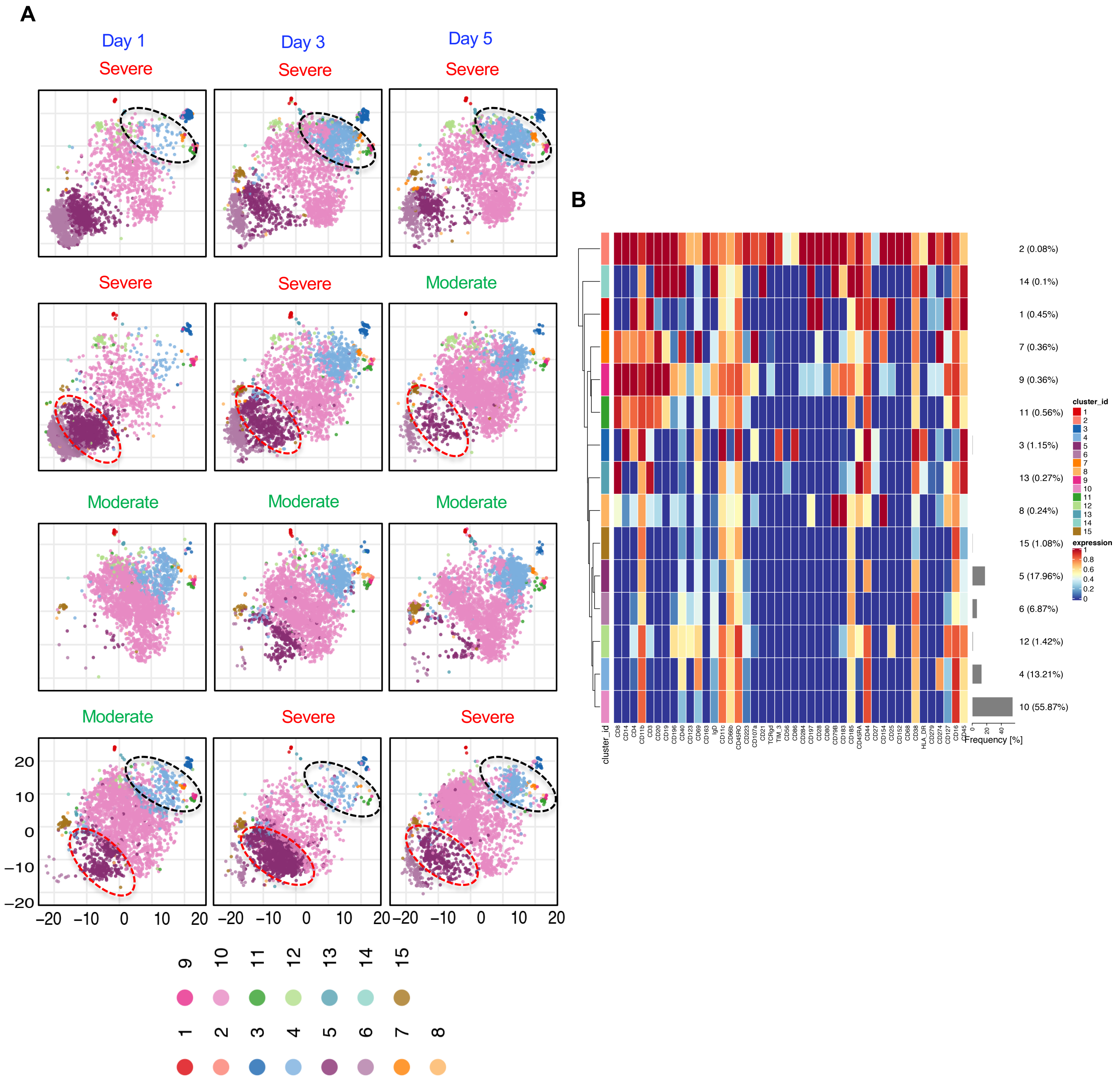

Morrissey et al, Supplemental Figure 4

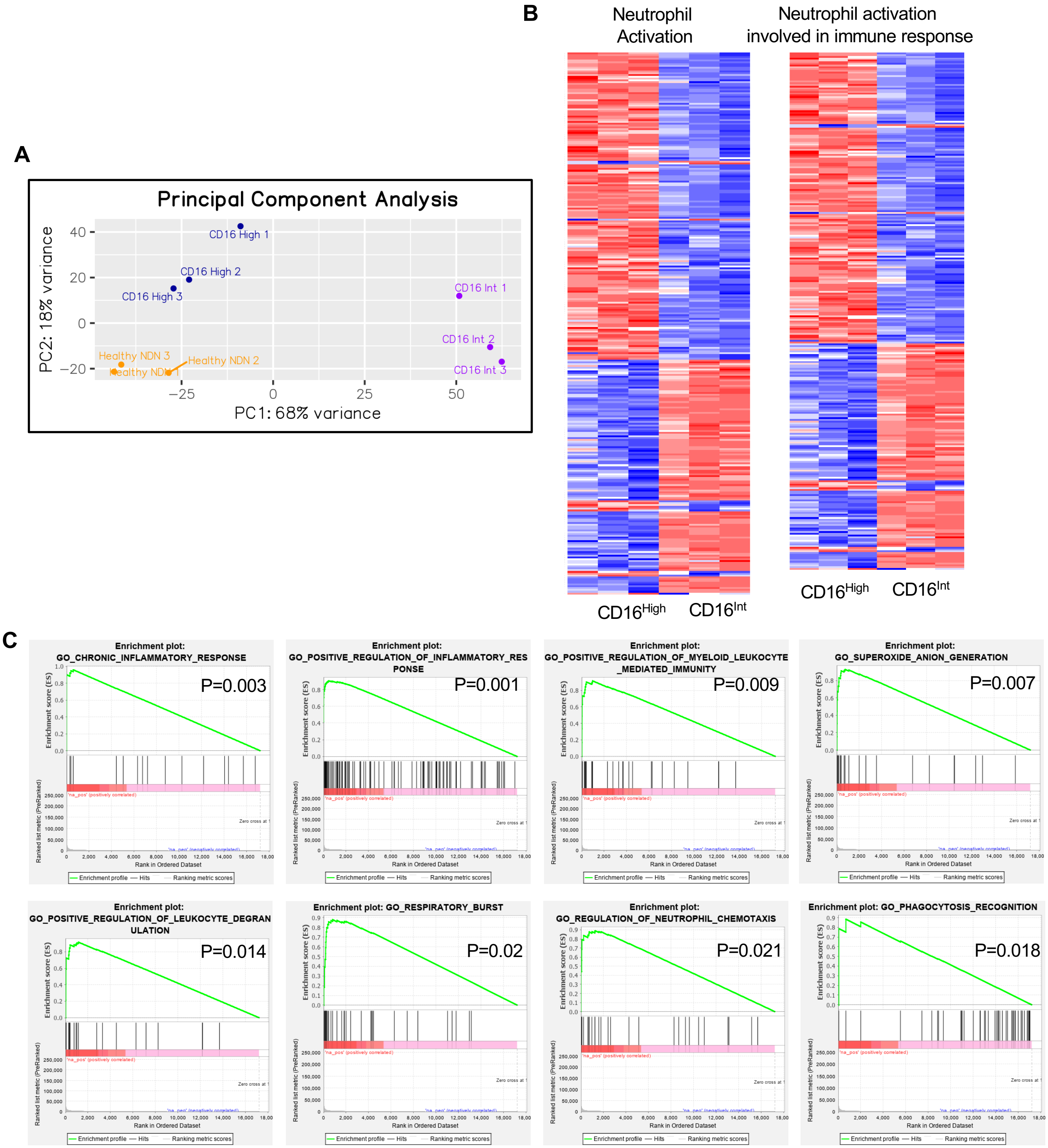

Morrissey et al, Supplemental Figure 5

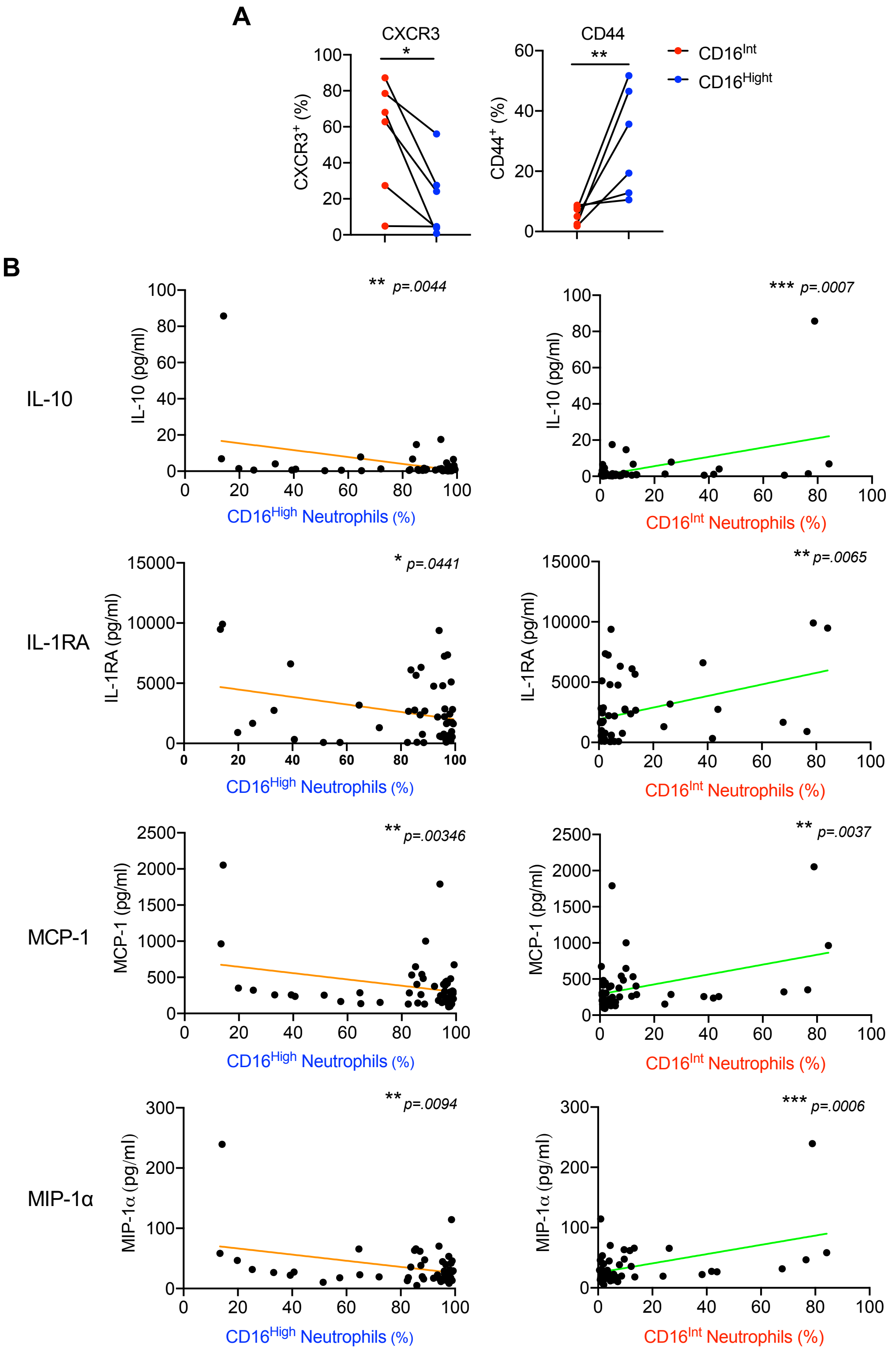

Morrissey et al, Supplemental Figure 6

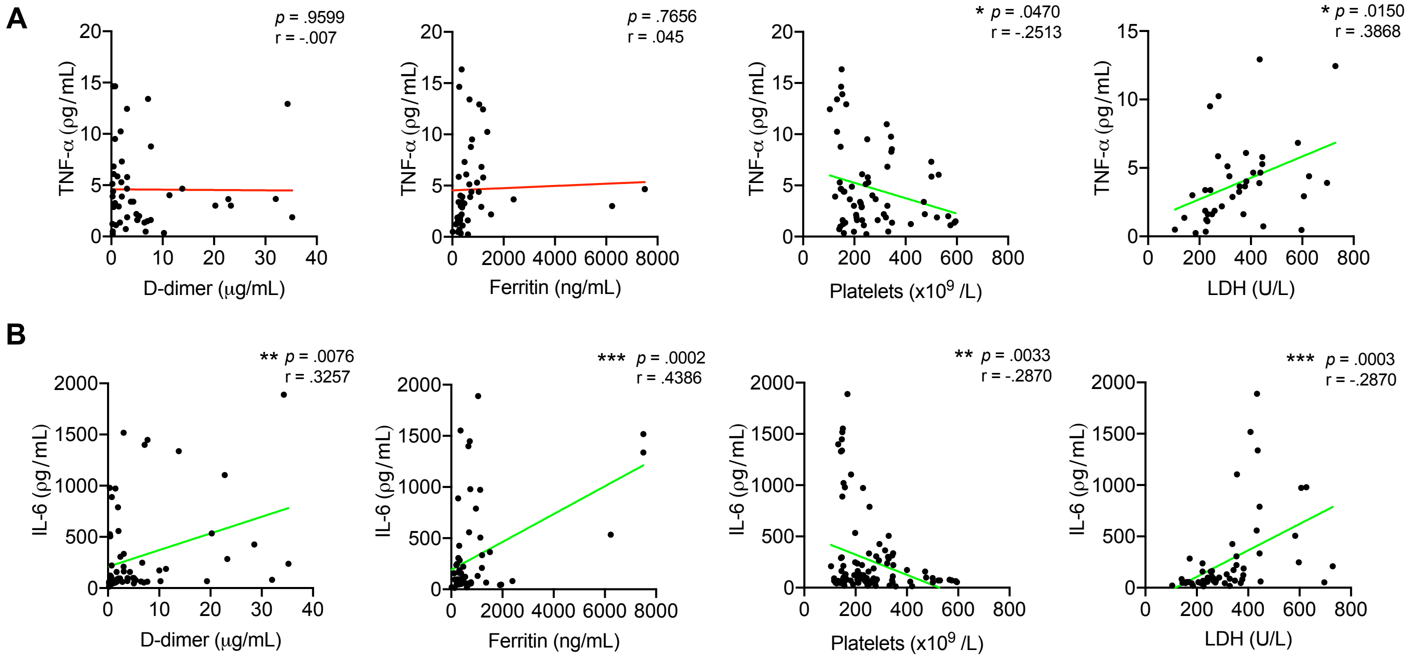

Morrissey et al, Supplemental Figure 7

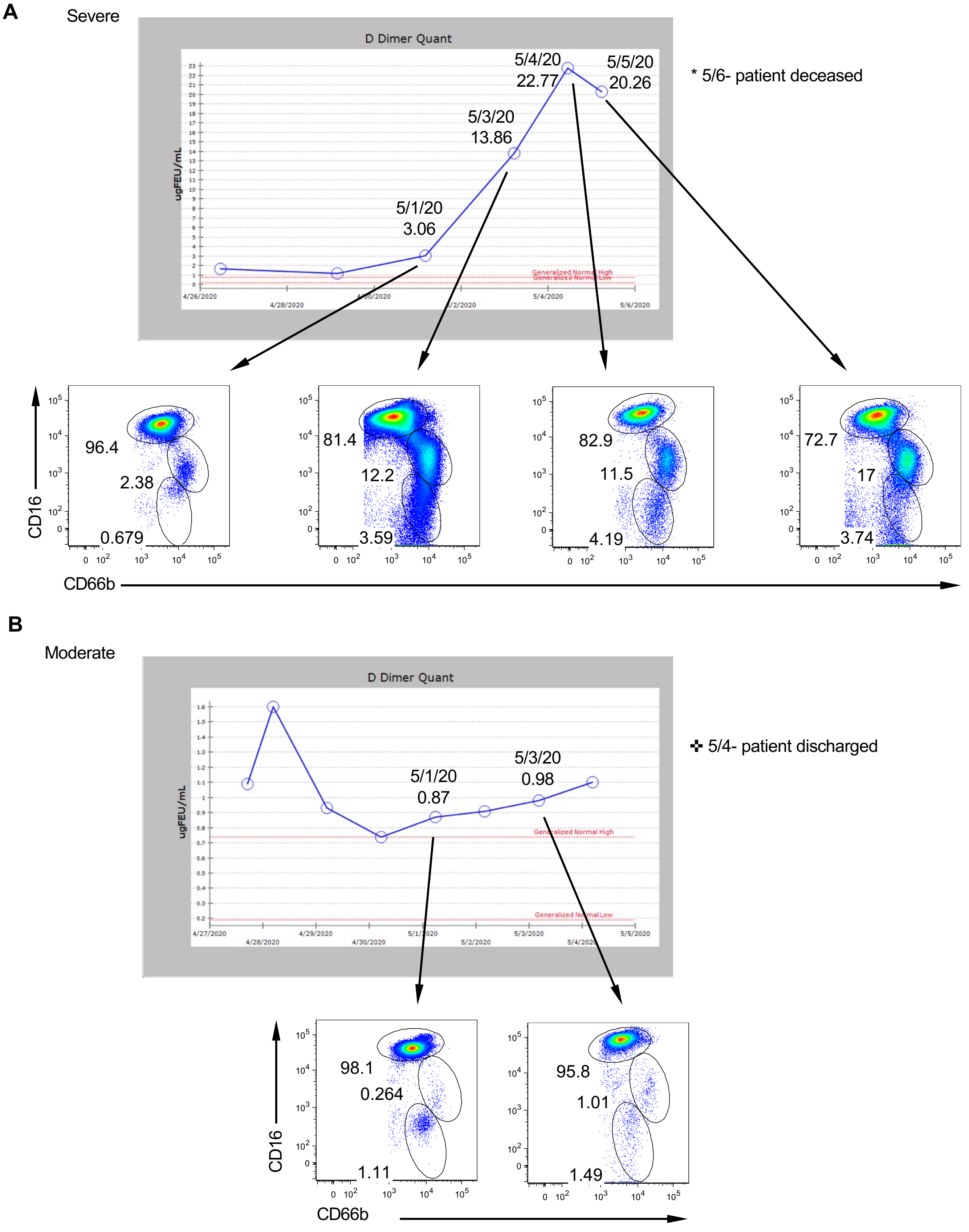

Supplement: Supplemental data [file jciinsight-6-148435-s085.pdf]
